# Modulation of Titin-Based Stiffness in Hypertrophic Cardiomyopathy via Protein Kinase D

**DOI:** 10.3389/fphys.2020.00240

**Published:** 2020-04-15

**Authors:** Melissa Herwig, Detmar Kolijn, Mária Lódi, Soraya Hölper, Árpád Kovács, Zoltán Papp, Kornelia Jaquet, Peter Haldenwang, Cris Dos Remedios, Peter H. Reusch, Andreas Mügge, Marcus Krüger, Jens Fielitz, Wolfgang A. Linke, Nazha Hamdani

**Affiliations:** ^1^Department of Molecular and Experimental Cardiology, Ruhr University Bochum, Bochum, Germany; ^2^Department of Cardiology, St. Josef-Hospital, Ruhr University Bochum, Bochums, Germany; ^3^Department of Clinical Pharmacology, Ruhr University Bochum, Bochum, Germany; ^4^Institute of Physiology, Ruhr University Bochum, Bochum, Germany; ^5^Division of Clinical Physiology, Department of Cardiology, Faculty of Medicine, University of Debrecen, Debrecen, Hungary; ^6^Kálmán Laki Doctoral School, University of Debrecen, Debrecen, Hungary; ^7^Sanofi-Aventis Deutschland GmbH Industriepark Höchst, Frankfurt, Germany; ^8^Department of Cardiothoracic Surgery, University Hospital Bergmannsheil Bochum, Bochum, Germany; ^9^School of Medical Sciences, Bosch Institute, University of Sydney, Camperdown, NSW, Australia; ^10^Institute for Genetics, Cologne Excellence Cluster on Cellular Stress Responses in Aging-Associated Diseases, Cologne, Germany; ^11^Center for Molecular Medicine (CMMC), University of Cologne, Cologne, Germany; ^12^Department of Internal Medicine B, Cardiology, University Medicine Greifswald, Greifswald, Germany; ^13^DZHK (German Center for Cardiovascular Research), Partner Site Greifswald, Greifswald, Germany; ^14^Institute of Physiology II, University Hospital Münster, University of Münster, Münster, Germany

**Keywords:** titin, HCM, PKD, Hsp27, stiffness

## Abstract

The giant protein titin performs structure-preserving functions in the sarcomere and is important for the passive stiffness (F_passive_) of cardiomyocytes. Protein kinase D (PKD) enzymes play crucial roles in regulating myocardial contraction, hypertrophy, and remodeling. PKD phosphorylates myofilament proteins, but it is not known whether the giant protein titin is also a PKD substrate. Here, we aimed to determine whether PKD phosphorylates titin and thereby modulates cardiomyocyte F_passive_ in normal and failing myocardium. The phosphorylation of titin was assessed in cardiomyocyte-specific PKD knock-out mice (cKO) and human hearts using immunoblotting with a phosphoserine/threonine and a phosphosite-specific titin antibody. PKD-dependent site-specific titin phosphorylation *in vivo* was quantified by mass spectrometry using stable isotope labeling by amino acids in cell culture (SILAC) of SILAC-labeled mouse heart protein lysates that were mixed with lysates isolated from hearts of either wild-type control (WT) or cKO mice. F_passive_ of single permeabilized cardiomyocytes was recorded before and after PKD and HSP27 administration. All-titin phosphorylation was reduced in cKO compared to WT hearts. Multiple conserved PKD-dependent phosphosites were identified within the Z-disk, A-band and M-band regions of titin by quantitative mass spectrometry, and many PKD-dependent phosphosites detected in the elastic titin I-band region were significantly decreased in cKO. Analysis of titin site-specific phosphorylation showed unaltered or upregulated phosphorylation in cKO compared to matched WT hearts. F_passive_ was elevated in cKO compared to WT cardiomyocytes and PKD administration lowered F_passive_ of WT and cKO cardiomyocytes. Cardiomyocytes from hypertrophic cardiomyopathy (HCM) patients showed higher F_passive_ compared to control hearts and significantly lower F_passive_ after PKD treatment. In addition, we found higher phosphorylation at CaMKII-dependent titin sites in HCM compared to control hearts. Expression and phosphorylation of HSP27, a substrate of PKD, were elevated in HCM hearts, which was associated with increased PKD expression and phosphorylation. The relocalization of HSP27 in HCM away from the sarcomeric Z-disk and I-band suggested that HSP27 failed to exert its protective action on titin extensibility. This protection could, however, be restored by administration of HSP27, which significantly reduced F_passive_ in HCM cardiomyocytes. These findings establish a previously unknown role for PKDin regulating diastolic passive properties of healthy and diseased hearts.

## Introduction

Protein kinase (PK)D is a serine/threonine kinase that belongs to the family of calcium/calmodulin-dependent kinases (CaMKII) due to its catalytic domain structure and substrate specificity. The PKD kinase family consists of three members: PKD1 (formerly known as PKCμ) (Valverde et al., 1994) and the predominant isoforms in the heart (Sin and Baillie, 2012), PKD2 (Sturany et al., 2001), and PKD3 (also known as PKCν) (Hayashi et al., 1999). The isoforms differ in structural and enzymatic properties from members of the PKC family. Some substrates that are targets of PKC are not phosphorylated by PKD (Johannes et al., 1994; Valverde et al., 1994; Van Lint et al., 1995), and unlike CaMKII, PKD is not directly activated by Ca^2+^ or calmodulin (Avkiran et al., 2008).

PKD can be activated by other stimuli including reactive oxygen species (ROS), growth factors (i.e., platelet-derived growth factor), and triggering of immune cell receptors. The PKD kinase is involved in the regulation of myocardial contraction by phosphorylating cardiac myosin binding protein C (cMyBP-C), cardiac troponin I (cTnI) and the L-type, voltage gated Ca^2+^ channel (Haworth et al., 2004; Cuello et al., 2007; Aita et al., 2011; Dirkx et al., 2012). Phosphorylation of TnI by PKD resulted in a significant rightward shift of the tension–pCa relationship, indicating reduced myofilament Ca^2+^ sensitivity (Haworth et al., 2004; Cuello et al., 2007; Aita et al., 2011; Dirkx et al., 2012). At submaximal Ca^2+^ activation, PKD-mediated phosphorylation also accelerated isometric cross-bridge cycling kinetics (Haworth et al., 2004; Cuello et al., 2007), suggesting a beneficial effect of PKD activation on cardiac function. PKD also alters gene expression leading to hypertrophy and influencing cardiac remodeling processes (Vega et al., 2004; Harrison et al., 2006).

With a molecular mass ranging from 3,000 to 3,700 kDa, titin is the largest known protein and its function as a molecular spring is important for the elasticity of striated muscle. Spanning the half-sarcomere from the Z-disk to the M-band, titin is expressed in various isoforms that confer different elastic properties to the sarcomere. Two main isoform classes of titin are expressed in the human heart: a shorter, stiffer N2B (3,000 kDa) isoform and several longer, more compliant, N2BA isoforms (>3,200 kDa). These variants differ in their elastic I-band region due to alternative splicing, whereas the Z-disk, A-band, and M-band regions of titin are essentially constitutively expressed. The titin spring region has a complex sequence comprising two types of extensible segments: (1) regions composed of immunoglobulin-like (Ig-) domains (proximal; middle; distal) arranged in tandem; and (2) intrinsically disordered structures, including a unique sequence of the cardiac specific “N2-B” element (“N2-Bus”) and the “PEVK” segment rich in proline, glutamate, valine, and lysine.

The titin filaments in cardiomyocytes are considered to be a main determinant of myocardial “passive” stiffness, along with the collagen fibers of the extracellular matrix and additional factors such as diastolic Ca^2+^ levels. We have previously demonstrated that the stiffness of cardiac titin is highly variable and depends on titin isoform switching and/or post-translational modifications such as phosphorylation and oxidation (Linke and Hamdani, 2014).

In our previous study, using a CaMKII knockout mouse model together with the stable isotope labeling of amino acids in cell culture (SILAC) mouse model, we found ~20 CaMKII-dependent phosphosites along the titin molecule (Hamdani et al., 2013b). Some phosphosites were located within the extensible region of titin, e.g., at the N2-Bus and PEVK spring elements, while others were within the A-band, M-band, and Z-disk regions of titin. Using this approach, we also detected >50 additional titin phosphosites that appeared to be not regulated by CaMKII. As regards to other kinases, protein kinase A and G and ERK2 were shown to phosphorylate the titin springs at specific sites within the cardiac-specific N2-Bus element (Kruger et al., 2009; Kotter et al., 2013). This modification alters the molecular stiffness of N2-Bus. Yet another kinase, PKCα, was shown to phosphorylate titin's PEVK element (Hidalgo et al., 2009).

A well-known substrate of PKD, among others, is heat shock protein 27 (HSP27) (Doppler et al., 2005). HSPs are molecular chaperones comprising a large family of proteins involved in protection against various forms of cellular stress (Mymrikov et al., 2011). HSPs generally facilitate protein refolding, target misfolded proteins to the proteasome and stabilize and transport partially folded proteins to different cellular compartments. Under various environmental insults such as heat, oxidative stress or acidosis, protein denaturation occurs and leads to unfolding and undesirable protein aggregates (Mymrikov et al., 2011). HSP27 is a small (ATP-independent) HSP whose function is regulated, in part, by posttranslational modifications, including phosphorylation at Ser-18, Ser-78, Ser-82, and Thr-143 (Kostenko and Moens, 2009; Mymrikov et al., 2011). Previous work showed that the unfolded Ig regions of I-band titin, but not the intrinsically disordered segments, can aggregate, causing high titin-dependent myocyte stiffness, and that HSP27 prevented this aggregation and suppressed the stiffening (Kotter et al., 2014). We speculated that cardiomyocyte elastic function could be protected under stress, at least in part, by HSP27 being regulated via PKD-mediated phosphorylation. The main aim of the present study was to investigate the effects of PKD on titin phosphorylation *in vivo* and resulting functional changes using cardiomyocyte specific *Prkd1*, which encodes for PKD1, knockout mice, SILAC mouse hearts, and human hypertrophic cardiomyopathy (HCM) heart tissues. We identified various PKD1-dependent phosphosites within titin using quantitative mass spectrometry (MS) and showed that PKD1-mediated titin-phosphorylation reduces cardiomyocyte F_passive_. Additionally, we found increased oxidative stress in human HCM tissues along with increased HSP27 expression and phosphorylation. HCM tissues also showed increased CaMKII-dependent phosphorylation at the PEVK and N2Bus titin regions. High cardiomyocyte stiffness was corrected by incubation with HSP27 and PKD, probably through relief of titin aggregation. Taken together, our results highlight the important roles of PKD and HSP27, in the presence of oxidative stress, in modulating diastolic function via titin-based stiffness regulation.

## Methods

Detailed methods descriptions are provided in the online Supplementary Methods.

### Human Heart Tissues

The investigation conforms to the principles outlined in the Declaration of Helsinki and samples were obtained after informed consent and with approval of the local Ethics Committee. LV tissue was obtained from end-stage heart failure patients (NYHA III or IV; *n* = 10), hypertrophic cardiomyopathy (male, mean age 45 years). LV tissue from non-failing donor hearts (*N* = 10; +- 40 years of age) served as reference and was obtained from donor hearts (*n* = 5).

### Cardiomyocyte Specific *Prkd1* Knock-Out Mice

All animal procedures were performed in accordance with the guidelines of Charité Universitätsmedizin Berlin as well as Max-Delbrück Center for Molecular Medicine and were approved by the Landesamt für Gesundheit und Soziales (LaGeSo, Berlin, Germany) for the use of laboratory animals (permit number: G 0229/11) and followed the ‘Principles of Laboratory Animal Care’ (NIH publication no. 86–23, revised 1985) as well as the current version of German Law on the Protection of Animals. The generation and usage of the conditional *Prkd1* allele was published elsewhere (Fielitz et al., 2008; Kim et al., 2008). The Cre-loxP recombination system was used for the generation of a conditional *Prkd1* allele. *Prkd1*loxP/loxP mice were crossed with Cre carrying mice controlled by cardiomyocyte-specific alpha-myosin-heavy-chain promotor (αMHC-Cre) (Agah et al., 1997) (cKO, *Prkd1*loxP/loxP; αMHC-Cre). αMHC-Cre-negative littermates were used as controls (WT, *Prkd1*loxP/loxP). Cardiac tissue was obtained when mice were 8–10 weeks of age. *N* = 7 for both KO and WT.

### SILAC-Based Quantitative Mass Spectrometry

We mixed equal amounts of protein lysates from heart tissue (7.5 mg) from the 13Lys6 heavy-labeled SILAC mouse and a non-labeled WT or non-labeled *Prkd1* cKO mouse. After protein digestion and phosphopeptide enrichment, the ratio of labeled:unlabeled peptides was determined by liquid chromatography and tandem MS and used to identify the cKO:WT ratio of titin phosphopeptides (Kruger et al., 2008).

### Titin Isoform Separation

Homogenized myocardial samples were analyzed by 1.8% SDS-PAGE. Protein bands were visualized by Coomassie staining and analyzed densitometrically.

### All-Titin Phosphorylation Assays

Titin bands were stained with anti-phospho-antibody directed against phospho-serine/threonine. Phospho-protein signals were indexed to total-protein signals and normalized to the intensity of coomassie staining to correct for differences in sample loading. Alternatively, all-titin phosphorylation was measured by PKD-mediated back-phosphorylation (Hamdani et al., 2013b).

### Titin and Phosphotitin Western Blots

Western blots were performed using custom-made, affinity-purified, anti-phosphoserine-specific antibodies directed against phospho-Ser-4010, -Ser-4062, -Ser-4099 (all N2Bus), -Ser-11878, and –Ser-12022 (both PEVK), of human titin (UniProtKB identifyer, Q8WZ42), and antibodies recognizing the corresponding nonphosphorylated sequence at these sites (Hamdani et al., 2013b). We also used phosphosite-specific antibodies against phospho-Ser-3991, -Ser-4043, -Ser-4080 (all N2Bus), -Ser-12742, and –Ser-12884 (both PEVK), of mouse titin (UniProtKB identifyer, A2ASS6) (Hamdani et al., 2013b).

### Force Measurements on Isolated Cardiomyocytes

Cardiomyocytes were skinned and single isolated cells (*n* = 12–42/5–6 heart/group) attached between a force transducer and motor (Hamdani et al., 2013a,b). F_passive_ was recorded over the sarcomere length (SL) range, 1.8–2.4 μm, and was measured before/after PKD and/or HSP27 incubation.

### Quantification of Tissue Oxidative Stress

Myocardial levels (*n* = 7 LV sample/group) of oxidative stress markers were tested with enzyme-linked immunosorbent assay (ELISA). Hydrogen peroxide (H_2_O_2_) was assessed in LV tissue homogenates (*n* = 4–10/group). Samples containing equal amounts of total protein were analyzed for H_2_O_2_ formation. Total reduced glutathione in heart samples was determined in duplicate with a colorimetric glutathione assay kit (CS0260, Sigma Aldrich).

### Amount and Phosphorylation of PKD and HSP27

The content of PKD and HSP27, as well as their phosphorylation were measured by 15% SDS–PAGE and western blot.

### CaMKII Content and Activity

The content of CaMKII was determined using 15% SDS–PAGE and western blot and its activity by using non-radioactive kinase activity-assay kit (CycLex).

### Immunofluorescence Imaging

Frozen histological LV sections (*n* = 3/group) were fixed, blocked and dual-stained with anti-PKD (Sigma-Aldrich; dilution 1:200) or anti-phospho-HSP27 (Ser 82) (Cell Signaling Technology; 1:50) and anti-α-actinin (sarcomere; Sigma-Aldrich; dilution 1:400) antibody, and were incubated with the appropriate secondary antibodies: (FITC) anti-mouse (Rockland Immunochemicals Inc, Limerick, PA, USA; dilution 1:300) and Cy3 anti-rabbit (Jackson ImmunoResearch Laboratories Inc, West Grove, PA, USA; dilution 1:100). Immunostained samples were analyzed by confocal laser scanning microscopy (Nikon Eclipse Ti-E Inverted Microscope System; Nikon Instruments, Nikon Corp, Shinagawa, Tokyo, Japan). Immunofluorescence imaging was processed equally among groups, for 2D intensity histogram analysis the Coloc2 plugin in FIJI was used.

### Electron Microscopy (EM)

Frozen LV samples were cut, fixed and blocked. Samples were then immunolabeled against primary antibodies of PKD (Abcam; 1:200) and HSP27 (Abcam; 1:200), then with nanogold conjugated secondary antibodies. After counterstaining with osmium-tetroxide, and dehydration, blocks were embedded into resin. 50 nm thin sections were cut.

### Statistics

Values are given as mean ± SEM. Statistically significant differences were tested using Bonferroni adjusted unpaired or paired Student's *t*-test, with *P* < 0.05 considered significant.

## Results

We hypothesized that *Prkd1* cKO mouse hearts would show altered titin phosphorylation compared to matched WT hearts.

### Quantitative Mass Spectrometry Detects Disturbed Regulation in *Prkd1* cKO Mice

**Figure 1:**
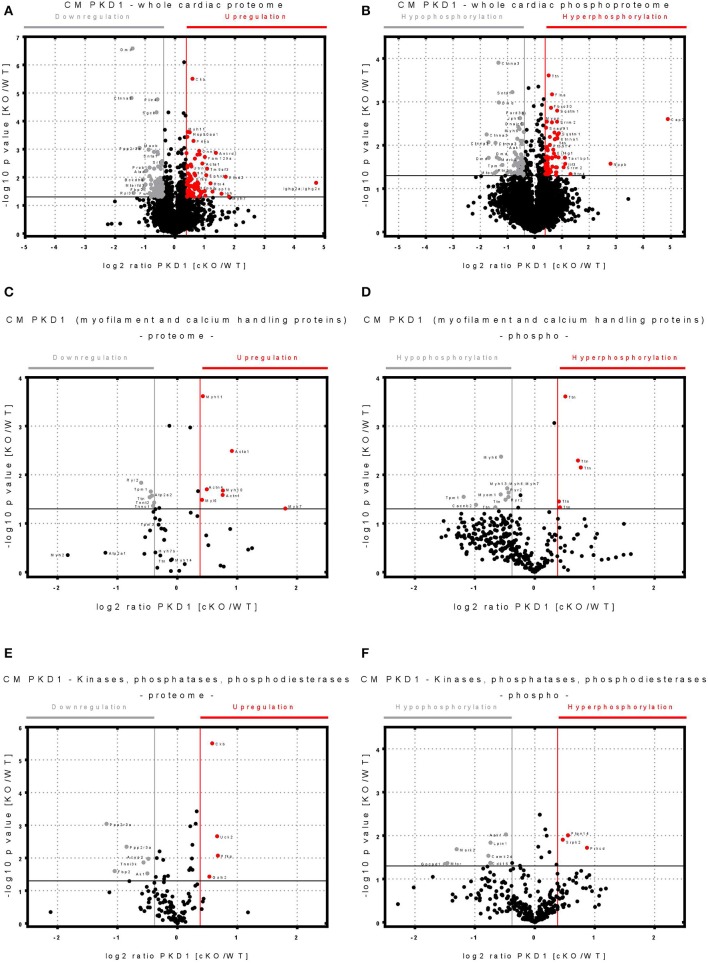
Volcano plots showing changes in the cardiac proteome and phosphoproteome in protein kinase D 1 (PKD1)-cKO and matched wild-type (WT) mouse hearts. **(A)** Volcano plot of whole cardiac proteome with identified significantly up- or down-regulated peptides in wild type (WT) vs. cardiomyocyte specific Protein Kinase D1 (PKD1) knock-out (KO) animals. **(B)** Volcano plot of whole cardiac phosphoproteome with identified significantly hyper- or hypo-phosphorylated peptides in WT vs. PKD1 cKO mice. **(C)** Volcano plot of myofilament and calcium handling proteins with significantly up- or down-regulated peptides in WT vs. PKD1 cKO mice. **(D)** Volcano plot of myofilament and calcium handling proteins with significantly hyper- or hypo-phosphorylated peptides in WT vs. PKD1 cKO mice. **(E)** Volcano plot of kinases, phosphatases, phosphodiesterases identified with significantly up- or down-regulated peptides in WT vs. PKD1 cKO mice. **(F)** Volcano plot of kinases, phosphatases, phosphodiesterases identified with significantly hyper- or hypo-phosphorylated peptides in WT vs. PKD1 cKO mice. Protein content or phosphorylation considered as significantly changed (-log10 *p* ≥ 1.3, log2 ratio ≥ ± 0.37) over the WT are color-coded. Only values with a—log10 *p* > 0 are shown. Experiments were performed in triplicates.

Using the SILAC technique, we detected a wide range of cardiac proteins that were either significantly upregulated (>80 proteins), downregulated (>105 proteins) or unchanged (remainder) in cKOs (Figure 1A). In total we detected 9,833 phosphosites (4,859 of them with −log10 *p* > 0) from 3,652 different proteins (2,507 of them with log10 *p* > 0) (Figure 1B). In total, 505 proteins were downregulated in cKO (log2 ratio KO/WT < −0.37) with a significant downregulation of 105 proteins. In addition to 422 proteins found to be upregulated (log2 ratio KO/WT >0.37), 80 proteins showed a significant upregulation, while the rest remained either unchanged or with no relevant pvalue (Figure 1A). Among the significantly up- or downregulated proteins were numerous myofilament and calcium handling proteins, in addition to several phosphatases and kinases (Figures 1C–F). We also detected many cardiac peptides that were either hypophosphorylated (70 phosphopeptides), hyperphosphorylated (63 phosphopeptides), or unchanged in cKOs (Figure 1B).

### Quantitative Mass Spectrometry Detects Conserved PKD Phosphosites in Titin

**Figure 2:**
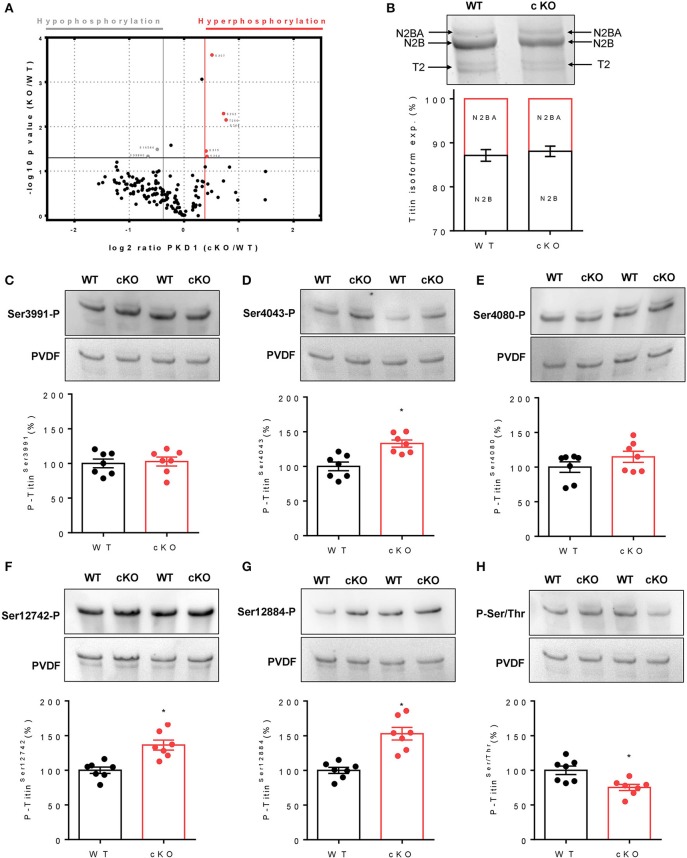
Titin phosphorylation and titin isoform composition in protein kinase D1 knockout (PKD1 cKO) and matched wild-type (WT) mouse hearts. **(A)** Volcano plot of SILAC-quantified titin phosphosites in heart tissue of PKD1 cKO vs. WT mice (*n* = 3 hearts/group). **(B)** Titin isoform composition. **(C)** Site-specific phosphorylation (P) of titin-N2B isoform at Ser3991 within N2Bus. **(D)** Site-specific P of titin-N2B isoform at Ser4043 within N2Bus. **(E)** Site-specific P of titin-N2B isoform at Ser4080 within N2Bus. **(F)** Site-specific P of titin-N2B isoform at Ser12742 within PEVK segment. **(G)** Site-specific P of titin-N2B isoform at Ser12884 within PEVK segment. **(H)** Total N2B-titin phosphorylation in PKD1 cKO/WT hearts. Western blot data are means ± SEM; *n* = 7 hearts/group; each heart analyzed in duplicate. *P < 0.05 in Students unpaired *t*-test.

Quantitative MS was combined with a modified SILAC technique to facilitate identification of PKD-dependent phosphorylation sites in titin. Titin was among the proteins that showed the largest alterations in phosphorylation in cKO compared to WT hearts. We identified 332 titin phosphopeptide (including 77.7% serines, 19.3% threonines, and 3.0% tyrosines), including 258 phosphosites with a ratio of cKO to WT phosphorylation levels (ratio WT/cKO) for titin. Among them, 164 phosphosites were with –log10 *p* > 0 (Figure 2A). Most of the identified phosphopeptides were class 1 (localization probability 0.75–1.00), although 27 phosphopeptides showed a localization probability <0.75 and were therefore class 2 phosphopeptides (see Tables 1–5). The majority of the phosphopeptides showed single phosphorylation, although some showed double phosphorylation. According to the conservative estimate that a cKO/WT ratio should be ≤ −0.37 or ≥ +0.37 in order to represent a significant change in cKO vs. WT, ten significantly altered titin phosphorylation sites could be identified in cKO hearts (Table 1). We identified 24 phosphosites in the elastic spring region of titin (see Table 2). Most of them were located in the N2B-element, PEVK region and distal Ig region. In total, hypophosphorylation (log2 ratio cKO/WT < −0.37) was seen in 133 titin phosphopeptides, the majority of which originated from A- and M-band titin (see Table 3). Furthermore, 25 titin phosphosites were hyperphosphorylated (log2 ratio cKO/WT >0.37; Table 4). One hundred and twelve titin phosphopeptides showed no significant differences in phosphorylation in cKO compared to WT (−0.37> cKO/ WT ratio < +0.37; see Table 5).

**Table 1:** Significantly changed titin phosphosites (–log 10 *p*-value KO/WT > 1.3).

**Position within titin**	**UniProt identifyer**	**Log2 ratio (KO/WT)**	**–Log 10 *p*-value KO/WT**	**Charge**	**Multiplicity**	**Localization probability**	**Classification of phosphosite**	**Sequence window**
S33880	A2ASS6	−0.659036	1.33033	3	1	1	Class 1	DLYYYRRRRRSLGDMSDEELLLPIDDYLAMK
S264	A2ASS6	0.419615	1.33152	2	2	1	Class 1	PHKTPPRIPPKPKSRSPTPPSIAAKAQLARQ
S315	A2ASS6	0.40279	1.45166	2	1	0.997856	Class 1	PSPVRSVSPAGRISTSPIRSVKSPLLIRKTQ
S16544	A2ASS6	−0.489219	1.48858	3	1	0.993809	Class 1	AEEEEPFSLPLTERLSINNSKQGESQLRIRD
S20332	A2ASS6	−0.239112	1.58029	2	1	1	Class 1	IIGYVVEMRPKIADASPDEGWKRCNAAAQLI
S264	A2ASS6	0.767076	2.14935	2	3	1	Class 1	PHKTPPRIPPKPKSRSPTPPSIAAKAQLARQ
T266	A2ASS6	0.767076	2.14935	2	3	1	Class 1	KTPPRIPPKPKSRSPTPPSIAAKAQLARQQS
S262	A2ASS6	0.720452	2.29218	2	3	1	Class 1	QLPHKTPPRIPPKPKSRSPTPPSIAAKAQLA
S322	A2ASS6	0.325277	3.06343	2	1	1	Class 1	SPAGRISTSPIRSVKSPLLIRKTQTTTMATG
S307	A2ASS6	0.509144	3.60898	2	2	1	Class 1	VRHVRAPTPSPVRSVSPAGRISTSPIRSVKS

Z-disk.

*A-band*.

M-band.

*Multiplicity contains information about the phosphorylation of the peptides (single. double. triply phosphorylated)*.

**Table 2:** Detected phosphosites in the elastic I band titin spring (–log 10 *p*-value KO/WT >0).

**Position within titin**	**UniProt identifyer**	**Log2 ratio (KO/WT)**	**–Log 10 *p*-value KO/WT**	**Charge**	**Multiplicity**	**Localization probability**	**Classification of phosphosite**	**Sequence window**
T9146	A2ASS6	−0.650309	0.429695	3	1	0.616703	Class 2	KERLIPPSFTKKLSETVEETEGNSFKLEGRV
S3286	A2ASS6	−0.938293	0.866542	2	1	0.851679	Class 1	RPQPKISWYKEEQLLSTGFKCKFLHDGQEYT
S3977	A2ASS6	−0.319098	0.36361	2	1	0.840288	Class 1	CTEGKILMASADTLKSTGQDVALRTEEGKSL
T13665	A2ASS6	−0.316873	0.41921	2	1	1	Class 1	CEVSREPKTFRWLKGTQEITGDDRFELIKDG
S13228	A2ASS6	−0.273069	0	2	1	0.993076	Class 1	DIPGEWKLKGELLRPSPTCEIKAEGGKRFLT
S9201	A2ASS6	−0.230981	0	2	1	1	Class 1	IMFKNNALLLQVKRASMADAGLYTCKATNDA
S9144	A2ASS6	−0.203903	0.163212	2	1	1	Class 1	NIKERLIPPSFTKKLSETVEETEGNSFKLEG
S4175	A2ASS6	−0.168399	0.396358	3	1	1	Class 1	HEEDKIDVQGGRDHLSDAQKVETVIEAEADS
T14268	A2ASS6	−0.16409	0.087451	2	1	0.99985	Class 1	VFTKNLANLEVSEGDTIKLVCEVSKPGAEVI
S9459	A2ASS6	−0.158813	0.124587	3	1	1	Class 1	DLRAMLKKTPALKKGSGEEEEIDIMELLKNV
S13204	A2ASS6	−0.0662166	0.089154	2	1	0.880947	Class 1	LKPIEDVTIYEKESASFDAEISEEDIPGEWK
S4018	A2ASS6	−0.018641	0	2	1	0.675564	Class 2	VLLKEEQSEVVAVPTSQTSKSEKEPEAIKGV
S3991	A2ASS6	−0.0149712	0.0107483	2	1	0.999502	Class 1	KSTGQDVALRTEEGKSLSFPLALEEKQVLLK
S3870	A2ASS6	−0.00516502	0.0056415	3	1	0.991661	Class 1	EPEGVFPGASSAAQVSPVTIKPLITLTAEPK
S14664	A2ASS6	0.0113125	0.0110327	2	1	1	Class 1	REIKEGKKYKFEKDGSIHRLIIKDCRLEDEC
S13764	A2ASS6	0.200173	0	2	1	1	Class 1	SWFKNDQRLHTSKRVSMHDEGKTHSITFKDL
S3676	A2ASS6	0.258327	0	4	1	0.878152	Class 1	EDMPLYTSVCYTIIHSPDGSGTFIVNDPQRG
S12678	A2ASS6	0.331543	0.65471	3	1	1	Class 1	IEKPKLKPRPPARPPSPPKEDVKEKMFQLKA
T10276	A2ASS6	0.395205	0	3	1	0.637793	Class 2	EVQKKVVTEEKIAIITQREESPPPAVPEIPK
S10281	A2ASS6	0.719508	0.777963	3	1	0.999995	Class 1	VVTEEKIAIITQREESPPPAVPEIPKKKVPE
S12871	A2ASS6	0.829341	1.09019	3	1	1	Class 1	AKPKGPIKGVAKKTPSPIEAERKKLRPGSGG
S12884	A2ASS6	0.94305	0.52056	3	1	1	Class 1	TPSPIEAERKKLRPGSGGEKPPDEAPFTYQL
S12871	A2ASS6	0.981417	0.3495	3	2	1	Class 1	AKPKGPIKGVAKKTPSPIEAERKKLRPGSGG
T12869	A2ASS6	0.981417	0.3495	3	2	1	Class 1	EAAKPKGPIKGVAKKTPSPIEAERKKLRPGS

Proximal Ig region.

N2B-element.

Middle Ig region.

PEVK.

Distal Ig domains.

**Table 3:** Downregulated titin phosphosites (log2 ratio KO/WT <−0.37).

**Position within titin**	**UniProt identifyer**	**Log2 ratio (KO/WT)**	**–Log 10 *p*-value KO/WT**	**Charge**	**Multiplicity**	**Localization probability**	**Classification of phosphosite**	**Sequence window**
S23560	A2ASS6	−0.372779	0	3	1	0.692852	Class 2	PGPPGNPRVLDTSRSSISIAWNKPIYDGGSE
S18225	A2ASS6	−0.376743	0.548646	2	1	0.5	Class 2	PPGPPFPKVTDWTKSSVDLEWSPPLKDGGSK
S30484	A2ASS6	−0.380153	0	2	1	0.999969	Class 1	TWKLEEMRLKETDRMSIATTKDRTTLTVKDS
S34451	A2ASS6	−0.384564	0.573934	2	1	0.999956	Class 1	TLTVQKARVIEKAVTSPPRVKSPEPRVKSPE
S33933	A2ASS6	−0.390718	0.578927	2	1	0.999999	Class 1	SRSPPRFELSSLRYSSPPAHVKVEDRRRDFR
S34808	A2ASS6	−0.390976	0.349281	2	1	1	Class 1	IAEEVKRSAAASLEKSIVHEEVTKTSQASEE
S26062	A2ASS6	−0.391391	0	3	1	0.992938	Class 1	KWSVVAESKVCNAVVSGLSSGQEYQFRVKAY
S21726	A2ASS6	−0.391912	0.585051	2	1	0.999998	Class 1	FLTEENKWQRVMKSLSLQYSTKDLKEGKEYT
T27227	A2ASS6	−0.392365	0	3	1	0.598045	Class 2	QATVAWKKDGQVLRETTRVNVASSKTVTTLS
S34476	A2ASS6	−0.399261	0.538309	2	1	1	Class 1	RVKSPETVKSPKRVKSPEPVTSHPKAVSPTE
S28859	A2ASS6	−0.403171	0.471662	2	1	0.990318	Class 1	EPVVAKNAFVTPGPPSIPEVTKITKNSMTVV
T4070	A2ASS6-3	−0.408659	0.331878	3	1	0.998519	Class 1	RELLSFPVEIQITAATPTPEQNKECRELFEL
S22271	A2ASS6	−0.42308	0	3	1	0.944504	Class 1	VSVLDVPGPPGPIEISNVSAEKATLTWTPPL
S1196	A2ASS6	−0.429651	0.300969	3	1	0.887311	Class 1	ALVKTQQEMLYQTQMSTFIQEPKVGEIAPGF
S29764	A2ASS6	−0.433024	0.621301	2	1	0.999999	Class 1	FGPEYFDGLVIKSGDSLRIKALVQGRPVPRV
S26804	A2ASS6	−0.433475	0	3	1	0.524536	Class 2	LYPPGPPSNPKVTDTSRSSVSLAWNKPIYDG
S22184	A2ASS6	−0.435231	0	2	1	0.999983	Class 1	LDPTIKDGLTVKAGDSIVLSAISILGKPLPK
S28389	A2ASS6	−0.440064	0	2	1	0.999873	Class 1	ESVTLKWEPPKYDGGSHVTNYIVLKRETSTA
S19152	A2ASS6	−0.442809	0	2	1	0.686958	Class 2	RKDVATAQWSPLSTTSKKKSHMAKHLTEGNQ
S34292	A2ASS6	−0.458658	1.00349	2	1	0.997471	Class 1	LTKTEAYAVSSFKRTSELEAASSVREVKSQM
S848	A2ASS6	−0.472572	0.731925	2	1	0.999987	Class 1	ASIAGSAIATLQKELSATSSTQKITKSVKAP
S19518	A2ASS6	−0.479462	0	3	1	0.999999	Class 1	DITENAATVSWTLPKSDGGSPITGYYVERRE
T4827	A2ASS6-3	−0.480703	0	3	2	0.682593	Class 2	VRDTKHKAQLVQSDSTTSMEVEEVTFNTVYE
S16544	A2ASS6	−0.489219	1.48858	3	1	0.993809	Class 1	AEEEEPFSLPLTERLSINNSKQGESQLRIRD
S29489	A2ASS6	−0.490109	0.49045	2	1	0.999929	Class 1	GGGEITCYSIEKREASQTNWKMVCSSVARTT
S35097	A2ASS6	−0.491363	0.979128	3	1	0.972789	Class 1	ESFVEMSSSSFMGKSSMTQLESSTSRMLKAG
S29997	A2ASS6	−0.496859	0.246525	3	1	0.999978	Class 1	KKSTRWVKVISKRPISETRFKVTGLVEGNEY
S23358	A2ASS6	−0.498498	0.499119	3	1	0.855607	Class 1	RPGPPEGPLAVSDVTSEKCVLSWLPPLDDGG
S262	A2ASS6	−0.501588	0	2	1	1	Class 1	QLPHKTPPRIPPKPKSRSPTPPSIAAKAQLA
S30452	A2ASS6	−0.513035	0.782215	2	1	0.968235	Class 1	DLTGITNQLITCKAGSTFTIDVPISGRPAPK
S16548	A2ASS6	−0.515562	0.899345	3	1	0.999832	Class 1	EPFSLPLTERLSINNSKQGESQLRIRDSLRP
S24061	A2ASS6	−0.515855	0.613502	2	1	0.97456	Class 1	SVTLSWEPPKYDGGSSINNYIVEKRDTSTTA
T23053	A2ASS6	−0.515985	0.758139	2	1	0.999999	Class 1	SVVANYPFKVPGPPGTPQVTAVTKDSMTISW
S1120	A2ASS6	−0.516292	0.722335	2	1	1	Class 1	CQIGGNPKPHVYWKKSGVPLTTGYRYKVSYN
S21895	A2ASS6	−0.522994	0.473161	2	1	1	Class 1	EAMTLKWGPPKDDGGSEITNYVLEKRDSVNN
S34756	A2ASS6	−0.525721	0.586585	2	1	1	Class 1	VSTQKTSEVTSQKKASAQEEISQKALTSEEI
S21162	A2ASS6	−0.54349	0.319895	2	1	0.836052	Class 1	VNRKDSGDYTITAENSSGSKSATIKLKVLDK
S34778	A2ASS6	−0.543969	0.489795	2	1	0.999964	Class 1	QKALTSEEIKMSEVKSHETLAIKEEASKVLI
T22513	A2ASS6	−0.551522	0	3	1	0.958776	Class 1	VEHQKVGDDAWIKDTTGTALRITQFVVPDLQ
T24135	A2ASS6	−0.561529	0.975063	2	1	1	Class 1	PVVAQYPFKVPGPPGTPFVTLASKDSMEVQW
T23980	A2ASS6	−0.573932	0.604136	3	1	0.631224	Class 2	PPAVTWHKDDIPLKQTTRVNAESTENNSLLT
S21730	A2ASS6	−0.574432	0.464884	2	1	0.798102	Class 1	ENKWQRVMKSLSLQYSTKDLKEGKEYTFRVS
S23609	A2ASS6	−0.587423	0	2	1	0.995346	Class 1	VTPPAGLKATSYTITSLIENQEYKIRIYAMN
S4650	A2ASS6-3	−0.606701	0	3	1	0.981316	Class 1	ALFQTPSADVEEANVSETGASVENGDKTFIS
T25062	A2ASS6	−0.625343	0	3	1	0.499999	Class 2	KPSISWTKDGMPLKQTTRINVTDSLDLTTLS
T25063	A2ASS6	−0.625343	0	3	1	0.499999	Class 2	PSISWTKDGMPLKQTTRINVTDSLDLTTLSI
S16620	A2ASS6	−0.62959	0.594816	3	1	0.999999	Class 1	DSVLCKWEPPLDDGGSEIINYTLEKKDKTKP
S25817	A2ASS6	−0.632049	0.301091	2	1	1	Class 1	VKPEDKLEAPELDLDSELRKGIVVRAGGSAR
S34207	A2ASS6	−0.63678	0	2	1	0.992242	Class 1	AEVKWYHNGVELQESSKIHYTNTSGVLTLEI
S20215	A2ASS6	−0.644393	0.578427	2	1	0.989323	Class 1	EMTVVWNAPEYDGGKSITGYYLEKKEKHAVR
T30560	A2ASS6	−0.645399	0.70904	2	1	1	Class 1	ESCVLSWTEPKDDGGTEITNYIVEKRESGTT
T9146	A2ASS6	−0.650309	0.429695	3	1	0.616703	Class 2	KERLIPPSFTKKLSETVEETEGNSFKLEGRV
S33880	A2ASS6	−0.659036	1.33033	3	1	1	Class 1	DLYYYRRRRRSLGDMSDEELLLPIDDYLAMK
S21163	A2ASS6	−0.662774	0	2	1	0.555375	Class 2	NRKDSGDYTITAENSSGSKSATIKLKVLDKP
S32940	A2ASS6	−0.669661	0.567747	3	1	0.991067	Class 1	DSVNLTWTEPASDGGSKVTNYIVEKCATTAE
T27381	A2ASS6	−0.676248	0.40741	2	1	0.987482	Class 1	AVVAEYPFSPPGPPGTPKVVHATKSTMVVSW
S22466	A2ASS6	−0.68697	0.37929	3	1	0.971535	Class 1	NPVLMKDVAYPPGPPSNAHVTDTTKKSASLA
S15406	A2ASS6	−0.717398	0.426932	2	1	1	Class 1	NSIFLTWDPPKNDGGSRIKGYIVEKCPRGSD
S24060	A2ASS6	−0.726107	0.454637	2	1	0.965865	Class 1	DSVTLSWEPPKYDGGSSINNYIVEKRDTSTT
S17508	A2ASS6	−0.743691	0.827831	3	1	0.854636	Class 1	PPGPPSCPEVKDKTKSSISLAWKPPAKDGGS
S22646	A2ASS6	−0.746741	0	3	1	0.999997	Class 1	TGKFVMTIENPAGKKSGFVNVRVLDTPGPVL
Y21461	A2ASS6	−0.750766	0	2	1	0.997236	Class 1	NTEYQFRVYAVNKIGYSDPSDVPDKHCPKDI
S34464	A2ASS6	−0.758656	0.611225	2	1	1	Class 1	VTSPPRVKSPEPRVKSPETVKSPKRVKSPEP
S19128	A2ASS6	−0.761375	0.661405	3	1	1	Class 1	DSCYLTWKEPLDDGGSVVTNYVVERKDVATA
S17509	A2ASS6	−0.765849	0.574468	2	1	0.499998	Class 2	PGPPSCPEVKDKTKSSISLAWKPPAKDGGSP
S34009	A2ASS6	−0.77042	0.791858	2	1	1	Class 1	LLRPVTTTQRLSEYKSELDYMSKEEKSKKKS
S30820	A2ASS6	−0.787034	0	3	1	0.779125	Class 1	VLAKNAAGVISKGSESTGPVTCRDEYAPPKA
S34653	A2ASS6	−0.82199	0.752192	2	1	0.95726	Class 1	SSKPVIVTGLRDTTVSSDSVAKFTIKVTGEP
S32936	A2ASS6	−0.824822	0	3	1	0.909829	Class 1	DVSRDSVNLTWTEPASDGGSKVTNYIVEKCA
T16910	A2ASS6	−0.831267	0.77835	3	1	1	Class 1	LDVSVKGGIQIMAGKTLRIPAEVTGRPVPTK
S15236	A2ASS6	−0.836399	0.546251	3	1	0.997816	Class 1	DQVLEEGDRVKMKTISAYAELVISPSERTDK
S21637	A2ASS6	−0.851016	0.751694	2	1	0.999673	Class 1	WSTVTTECSKTSFRVSNLEEGKSYFFRVFAE
T30103	A2ASS6	−0.851493	0	3	1	0.499919	Class 2	DWHKVNTEPCVKTRYTVTDLQAGEEYKFRVS
S32230	A2ASS6	−0.864269	0.82444	2	1	0.991873	Class 1	DPFDKPSQPGELEILSISKDSVTLQWEKPEC
S34005	A2ASS6	−0.876525	0	3	1	0.999798	Class 1	EEEELLRPVTTTQRLSEYKSELDYMSKEEKS
S18731	A2ASS6	−0.87685	0.25695	3	1	0.995523	Class 1	EYMVISWKPPLDDGGSEITNYIIEKKELGKD
S4248	A2ASS6-3	−0.893375	0	4	1	0.967119	Class 1	VQGEPVRTHFYDHTVSPFAAQSNIKEYTIRE
T30453	A2ASS6	−0.895531	0.674399	3	1	0.861201	Class 1	LTGITNQLITCKAGSTFTIDVPISGRPAPKV
S1805	A2ASS6	−0.895653	0.717555	2	1	1	Class 1	GTDHTSATLIVKDEKSLVEESQLPDGKKGLQ
S15266	A2ASS6	−0.907094	0	2	1	0.977731	Class 1	KGIYTLTLENPVKSISGEINVNVIAPPSAPK
S25399	A2ASS6	−0.923229	0.531448	2	1	0.967678	Class 1	VIAKNAAGAISKPSDSTGPITAKDEVELPRI
S27960	A2ASS6	−0.925189	0.489628	2	1	0.834475	Class 1	RVRSLNKMGASDPSDSSDPQVAKEREEEPVF
S3286	A2ASS6	−0.938293	0.866542	2	1	0.851679	Class 1	RPQPKISWYKEEQLLSTGFKCKFLHDGQEYT
T22529	A2ASS6	−0.943427	0.67514	2	1	1	Class 1	GTALRITQFVVPDLQTKEKYNFRISAINDAG
S4720	A2ASS6-3	−0.959846	0	3	1	0.9499	Class 1	PRGAVHGAEVPHRRLSLSQDLPFLMTGEQQD
S35060	A2ASS6	−0.967557	0	2	1	0.845469	Class 1	SASKQEASFSSFSSSSASSMTEMKFASMSAQ
Y20757	A2ASS6	−0.96841	0.688841	2	1	0.999745	Class 1	SSVLIIKDVTRKDSGYYSLTAENSSGSDTQK
S25797	A2ASS6	−0.975471	0.492227	2	1	0.999986	Class 1	IRVCALNKVGLGEAASVPGTVKPEDKLEAPE
S23925	A2ASS6	−1.00025	0.887458	3	1	0.999906	Class 1	VSAQNEKGISDPRQLSVPVIAKDLVIPPAFK
S25613	A2ASS6	−1.02282	0.946511	2	1	1	Class 1	PVLMKNPFVLPGPPKSLEVTNIAKDSMTVCW
S29299	A2ASS6	−1.03903	0.614927	2	1	0.999999	Class 1	TDYLVERKGKGEQAWSHAGISKTCEIEIGQL
Y20182	A2ASS6	−1.04142	0.707871	3	1	1	Class 1	TGPPTESKPVIAKTKYDRPGRPDPPEVTKVS
S35104	A2ASS6	−1.0585	0	2	1	0.746582	Class 2	SSSFMGKSSMTQLESSTSRMLKAGGRGIPPK
S15616	A2ASS6	−1.06363	0.707178	2	1	0.999998	Class 1	GSKITNYVVERKATDSDVWHKLSSTVKDTNF
S20732	A2ASS6	−1.08258	0.581344	2	1	0.997277	Class 1	PICKWKKGDDEVVTSSHLAIHKADGSSVLII
T16946	A2ASS6	−1.0848	0	2	1	1	Class 1	EGELDKERVIIENVGTKSELIIKNALRKDHG
S24436	A2ASS6	−1.0957	0.769352	3	1	0.997797	Class 1	KVLDRPGPPEGPVAISGVTAEKCTLAWKPPL
S19776	A2ASS6	−1.13133	0	4	1	0.570984	Class 2	VRADHGKYIISAKNSSGHAQGSAIVNVLDRP
T30100	A2ASS6	−1.14081	0.86237	2	1	0.999845	Class 1	DLGDWHKVNTEPCVKTRYTVTDLQAGEEYKF
S28970	A2ASS6	−1.14229	0.390143	2	1	0.848484	Class 1	PPGPPAKIRIADSTKSSITLGWSKPVYDGGS
S23728	A2ASS6	−1.15678	0	2	1	0.680757	Class 2	FDSGKYILTVENSSGSKSAFVNVRVLDTPGP
S23604	A2ASS6	−1.18392	0	2	1	0.760984	Class 1	DEWQVVTPPAGLKATSYTITSLIENQEYKIR
T22805	A2ASS6	−1.18392	0.725798	2	1	0.948992	Class 1	IEAQRKGSDQWTHISTVKGLECVVRNLTEGE
S25730	A2ASS6	−1.2141	1.02176	2	1	0.999999	Class 1	AHVVDTTKNSITLAWSKPIYDGGSEILGYVV
S24155	A2ASS6	−1.21435	0.495954	3	1	0.938049	Class 1	LASKDSMEVQWHEPVSDGGSKVIGYHLERKE
T23603	A2ASS6	−1.22489	0.565233	2	1	0.891141	Class 1	EDEWQVVTPPAGLKATSYTITSLIENQEYKI
S29402	A2ASS6	−1.22605	1.20053	2	1	1	Class 1	LKDGLPLKESEYVRFSKTENKITLSIKNSKK
T25518	A2ASS6	−1.23095	0.735191	3	1	0.981598	Class 1	KVLDRPGPPEGPVQVTGVTAEKCTLAWSPPL
T19879	A2ASS6	−1.23865	1.098	3	1	1	Class 1	CAENKVGVGPTIETKTPILAINPIDRPGEPE
Y29398	A2ASS6	−1.26528	0.758789	2	1	1	Class 1	SISWLKDGLPLKESEYVRFSKTENKITLSIK
S32140	A2ASS6	−1.27419	0.799964	2	1	0.998818	Class 1	PEVLDVTKSSVSLSWSRPKDDGGSRVTGYYI
S28985	A2ASS6	−1.28346	0	2	1	0.999999	Class 1	SSITLGWSKPVYDGGSDVTGYVVEMKQGDEE
S35029	A2ASS6	−1.33441	0	3	1	0.960754	Class 1	PLVEEPPREVVLKTSSDVSLHGSVSSQSVQM
S34457	A2ASS6	−1.40297	0	2	2	1	Class 1	ARVIEKAVTSPPRVKSPEPRVKSPETVKSPK
S35096	A2ASS6	−1.44254	0.872837	2	1	0.869587	Class 1	QESFVEMSSSSFMGKSSMTQLESSTSRMLKA
T22515	A2ASS6	−1.45339	0	3	1	0.998937	Class 1	HQKVGDDAWIKDTTGTALRITQFVVPDLQTK
S25026	A2ASS6	−1.48484	0.743171	3	1	0.949735	Class 1	IAKDLVIEPDVRPAFSSYSVQVGQDLKIEVP
S21724	A2ASS6	−1.49957	0	2	1	0.965611	Class 1	VDFLTEENKWQRVMKSLSLQYSTKDLKEGKE
S27185	A2ASS6	−1.55785	0.714422	2	1	1	Class 1	QLGVPVIAKDIEIKPSVELPFNTFNVKANDQ
S34009	A2ASS6	−2.60171	0	2	2	1	Class 1	LLRPVTTTQRLSEYKSELDYMSKEEKSKKKS

Novex-3 (A2ASS6-3).

Z-disk.

Proximal Ig region.

Middle Ig region.

*A-band*.

M-band.

**Table 4:** Upregulated titin phosphosites (log2 ratio KO/WT > 0.37).

**Position within titin**	**UniProt identifyer**	**Log2 ratio (KO/WT)**	**–Log 10 p-value KO/WT**	**Charge**	**Multiplicity**	**Localization probability**	**Classification of phosphosite**	**Sequence window**
T314	A2ASS6	0.386936	1.09573	2	1	0.982868	Class 1	TPSPVRSVSPAGRISTSPIRSVKSPLLIRKT
T10276	A2ASS6	0.395205	0	3	1	0.637793	Class 2	EVQKKVVTEEKIAIITQREESPPPAVPEIPK
S315	A2ASS6	0.40279	1.45166	2	1	0.997856	Class 1	PSPVRSVSPAGRISTSPIRSVKSPLLIRKTQ
S264	A2ASS6	0.419615	1.33152	2	2	1	Class 1	PHKTPPRIPPKPKSRSPTPPSIAAKAQLARQ
S35038	A2ASS6	0.429709	0.499475	2	1	0.987556	Class 1	VVLKTSSDVSLHGSVSSQSVQMSASKQEASF
S262	A2ASS6	0.433659	0.711028	2	2	1	Class 1	QLPHKTPPRIPPKPKSRSPTPPSIAAKAQLA
S23788	A2ASS6	0.438542	0	2	1	0.993369	Class 1	KNYIVEKRESTRKAYSTVATNCHKTSWKVDQ
S1527	A2ASS6	0.44247	0	3	1	0.86319	Class 1	VIKEDGTQSLIIVPASPSDSGEWTVVAQNRA
S31025	A2ASS6	0.474799	0	3	1	0.838063	Class 1	GLGVPVESEPIVARNSFTIPSQPGIPEEVGA
S834	A2ASS6	0.503759	0.543576	2	1	0.99992	Class 1	VSKISVPKTEHGYEASIAGSAIATLQKELSA
S307	A2ASS6	0.509144	3.60898	2	2	1	Class 1	VRHVRAPTPSPVRSVSPAGRISTSPIRSVKS
T314	A2ASS6	0.608677	0	2	2	0.982868	Class 1	TPSPVRSVSPAGRISTSPIRSVKSPLLIRKT
S774	A2ASS6	0.620881	0.40983	2	1	0.98142	Class 1	HVVPQAVKPAVIQAPSETHIKTTDQMGMHIS
S879	A2ASS6	0.704194	0	3	2	0.998042	Class 1	TVKPGETRVRAEPTPSPQFPFADMPPPDTYK
S10281	A2ASS6	0.719508	0.777963	3	1	0.999995	Class 1	VVTEEKIAIITQREESPPPAVPEIPKKKVPE
S262	A2ASS6	0.720452	2.29218	2	3	1	Class 1	QLPHKTPPRIPPKPKSRSPTPPSIAAKAQLA
S264	A2ASS6	0.767076	2.14935	2	3	1	Class 1	PHKTPPRIPPKPKSRSPTPPSIAAKAQLARQ
T266	A2ASS6	0.767076	2.14935	2	3	1	Class 1	KTPPRIPPKPKSRSPTPPSIAAKAQLARQQS
S799	A2ASS6	0.787488	0	3	1	0.834492	Class 1	MGMHISSQVKKTTDISTERLVHVDKRPRTAS
S12871	A2ASS6	0.829341	1.09019	3	1	1	Class 1	AKPKGPIKGVAKKTPSPIEAERKKLRPGSGG
S12884	A2ASS6	0.94305	0.52056	3	1	1	Class 1	TPSPIEAERKKLRPGSGGEKPPDEAPFTYQL
S12871	A2ASS6	0.981417	0.3495	3	2	1	Class 1	AKPKGPIKGVAKKTPSPIEAERKKLRPGSGG
T12869	A2ASS6	0.981417	0.3495	3	2	1	Class 1	EAAKPKGPIKGVAKKTPSPIEAERKKLRPGS
S838	A2ASS6	1.20302	0	3	1	0.999952	Class 1	SVPKTEHGYEASIAGSAIATLQKELSATSST
S32133	A2ASS6	1.4809	0.355789	2	1	0.791719	Class 1	PEPPSNPPEVLDVTKSSVSLSWSRPKDDGGS

Z-disk.

PEVK.

**A-band**.

M-band.

**Table 5:** Unchanged titin phosphosites (−0.37 < log2 ratio KO/WT < 0.37).

**Position within titin**	**UniProt identifyer**	**Log2 ratio (KO/WT)**	**–Log 10 *p*-value KO/WT**	**Charge**	**Multiplicity**	**Localization probability**	**Classification of phosphosite**	**Sequence window**
T26299	A2ASS6	−0.367305	0.338896	2	1	0.999988	Class 1	PVVVQYPFKEPGPPGTPFVTSISKDQMLVQW
S23354	A2ASS6	−0.359797	0	3	1	0.990613	Class 1	KVLDRPGPPEGPLAVSDVTSEKCVLSWLPPL
S34298	A2ASS6	−0.356955	0	3	1	0.609727	Class 2	YAVSSFKRTSELEAASSVREVKSQMTETRES
S34470	A2ASS6	−0.355278	0	2	1	1	Class 1	VKSPEPRVKSPETVKSPKRVKSPEPVTSHPK
S33875	A2ASS6	−0.353589	0.477593	3	2	1	Class 1	PSPDYDLYYYRRRRRSLGDMSDEELLLPIDD
S33880	A2ASS6	−0.353589	0.477593	3	2	1	Class 1	DLYYYRRRRRSLGDMSDEELLLPIDDYLAMK
S18562	A2ASS6	−0.350673	0.345871	2	1	1	Class 1	IGTEKFHKVTNDNLLSRKYTVKGLKEGDTYE
T22520	A2ASS6	−0.329784	0	3	1	0.995336	Class 1	DDAWIKDTTGTALRITQFVVPDLQTKEKYNF
S3977	A2ASS6	−0.319098	0.36361	2	1	0.840288	Class 1	CTEGKILMASADTLKSTGQDVALRTEEGKSL
S19146	A2ASS6	−0.318534	0.168528	2	1	0.995484	Class 1	TNYVVERKDVATAQWSPLSTTSKKKSHMAKH
T13665	A2ASS6	−0.316873	0.41921	2	1	1	Class 1	CEVSREPKTFRWLKGTQEITGDDRFELIKDG
S17098	A2ASS6	−0.305806	0.472482	2	1	0.933227	Class 1	PPTSPERLTYTERTKSTITLDWKEPRSDGGS
S24868	A2ASS6	−0.302999	0	2	1	0.998408	Class 1	KIKNYIVEKREATRKSYAAVVTNCHKNSWKI
S34470	A2ASS6	−0.302672	0.238728	2	2	1	Class 1	VKSPEPRVKSPETVKSPKRVKSPEPVTSHPK
S26806	A2ASS6	−0.301597	0	2	1	0.655766	Class 2	PPGPPSNPKVTDTSRSSVSLAWNKPIYDGGA
S18224	A2ASS6	−0.300622	0.391952	2	1	0.832005	Class 1	APPGPPFPKVTDWTKSSVDLEWSPPLKDGGS
S34464	A2ASS6	−0.2992	0.235518	2	2	1	Class 1	VTSPPRVKSPEPRVKSPETVKSPKRVKSPEP
S2080	A2ASS6	−0.298846	0.520376	2	1	1	Class 1	ITIPTFKPERIELSPSMEAPKIFERIQSQTV
S19448	A2ASS6	−0.273899	0	2	1	0.805061	Class 1	QDTRKGTWGVVSAGSSKLKLKVPHLQKGCEY
S13228	A2ASS6	−0.273069	0	2	1	0.993076	Class 1	DIPGEWKLKGELLRPSPTCEIKAEGGKRFLT
S27374	A2ASS6	−0.266139	0	3	1	0.820467	Class 1	SSYSESSAVVAEYPFSPPGPPGTPKVVHATK
S20517	A2ASS6	−0.265893	0	3	1	0.748614	Class 2	TSCHVSWAPPENDGGSQVTHYIVEKREAERK
S28343	A2ASS6	−0.263658	0	3	1	0.780076	Class 1	SVTTDAGRYEITAANSSGTTKTFINIIVLDR
S32318	A2ASS6	−0.262435	0.516907	2	1	0.999971	Class 1	SRPRRTAMSVKTKLTSGEAPGVRKEMADVTT
S3622	A2ASS6-3	−0.260512	0	3	1	0.962608	Class 1	VEEKGMVRTIHFRSASPVRRADYVYNDEWSE
Y21274	A2ASS6	−0.257528	0	3	1	1	Class 1	VGDPILTEPAIAKNPYDPPGRCDPPVISNIT
T22220	A2ASS6	−0.252516	0.8886	3	1	0.940036	Class 1	AGKDIRPSDIAQITSTPTSSMLTVKYATRKD
S22152	A2ASS6	−0.241103	0	2	1	0.761358	Class 1	IRAKNTAGAISAPSESTGTIICKDEYEAPTI
S20332	A2ASS6	−0.239112	1.58029	2	1	1	Class 1	IIGYVVEMRPKIADASPDEGWKRCNAAAQLI
S9201	A2ASS6	−0.230981	0	2	1	1	Class 1	IMFKNNALLLQVKRASMADAGLYTCKATNDA
S4098	A2ASS6-3	−0.229673	0	3	1	1	Class 1	FELEPEVTPRDQAIQSPKHKFIFSSDITNEP
S27782	A2ASS6	−0.229019	0	3	1	0.547507	Class 2	DPFTTPSPPTSLEITSVTKDSMTLCWSRPET
T27228	A2ASS6	−0.228542	0.209869	3	1	0.955571	Class 1	ATVAWKKDGQVLRETTRVNVASSKTVTTLSI
S20036	A2ASS6	−0.211545	0	2	1	0.999133	Class 1	EVAWTKDKDATDLTRSPRVKIDTSAESSKFS
S9144	A2ASS6	−0.203903	0.163212	2	1	1	Class 1	NIKERLIPPSFTKKLSETVEETEGNSFKLEG
S22416	A2ASS6	−0.192457	0	2	1	0.999998	Class 1	EKKGLRWVRATKTPVSDLRCKVTGLQEGNTY
S30939	A2ASS6	−0.186488	0	2	1	0.998923	Class 1	VKVLDSPGPCGKLTVSRVTEEKCTLAWSLPQ
S18535	A2ASS6	−0.184072	0	3	1	1	Class 1	NTVSLTWNPPKYDGGSEIINYVLESRLIGTE
T266	A2ASS6	−0.174257	0.297613	2	1	1	Class 1	KTPPRIPPKPKSRSPTPPSIAAKAQLARQQS
S4175	A2ASS6	−0.168399	0.396358	3	1	1	Class 1	HEEDKIDVQGGRDHLSDAQKVETVIEAEADS
T23981	A2ASS6	−0.166376	0	3	1	0.5	Class 2	PAVTWHKDDIPLKQTTRVNAESTENNSLLTI
T14268	A2ASS6	−0.16409	0.087451	2	1	0.99985	Class 1	VFTKNLANLEVSEGDTIKLVCEVSKPGAEVI
S1529	A2ASS6	−0.159512	0.439196	3	1	0.943823	Class 1	KEDGTQSLIIVPASPSDSGEWTVVAQNRAGK
S9459	A2ASS6	−0.158813	0.124587	3	1	1	Class 1	DLRAMLKKTPALKKGSGEEEEIDIMELLKNV
T30575	A2ASS6	−0.154592	0	3	1	0.788648	Class 1	TEITNYIVEKRESGTTAWQLINSSVKRTQIK
S28281	A2ASS6	−0.146693	0.305079	3	1	0.999999	Class 1	DMKNFPSHTVYVRAGSNLKVDIPISGKPLPK
S26695	A2ASS6	−0.140014	0.346765	3	1	0.797368	Class 1	EPVIACNPYKRPGPPSTPEASAITKDSMVLT
S24391	A2ASS6	−0.137242	0.088548	2	1	0.993652	Class 1	TARLEIKSTDFATSLSVKDAVRVDSGNYILK
T29649	A2ASS6	−0.134127	0.58377	3	1	0.997724	Class 1	PSKFTLAVSPVDPPGTPDYIDVTRETITLKW
T21731	A2ASS6	−0.127027	0	2	1	0.718367	Class 2	NKWQRVMKSLSLQYSTKDLKEGKEYTFRVSA
T33772	A2ASS6	−0.120166	0.177658	2	1	1	Class 1	RMPYEVPEPRRFKQATVEEDQRIKQFVPMSD
S16477	A2ASS6	−0.120059	0.290657	2	1	0.960016	Class 1	KAVDPIDAPKVILRTSLEVKRGDEIALDATI
T25842	A2ASS6	−0.117083	0	2	1	0.999999	Class 1	AGGSARIHIPFKGRPTPEITWSKEEGEFTDK
S17015	A2ASS6	−0.116476	0	3	1	0.999637	Class 1	KMCLLNWSDPADDGGSDITGFIIERKDAKMH
S3827	A2ASS6-3	−0.110284	0.69199	2	1	1	Class 1	SNEEVHGYKSRGICESPDKVSQVLTPYPSES
S34488	A2ASS6	−0.108085	0.264442	2	1	0.999994	Class 1	RVKSPEPVTSHPKAVSPTETKPTEKGQHLPV
S790	A2ASS6	−0.10482	0	3	1	0.790373	Class 1	ETHIKTTDQMGMHISSQVKKTTDISTERLVH
T26696	A2ASS6	−0.0857946	0.182593	3	1	0.989467	Class 1	PVIACNPYKRPGPPSTPEASAITKDSMVLTW
S1977	A2ASS6	−0.081692	0.055874	2	1	0.990373	Class 1	KLQFEVQKVDRPVDTSETKEVVKLKRAERIT
S19447	A2ASS6	−0.0663668	0	2	1	0.591546	Class 2	KQDTRKGTWGVVSAGSSKLKLKVPHLQKGCE
S13204	A2ASS6	−0.0662166	0.089154	2	1	0.880947	Class 1	LKPIEDVTIYEKESASFDAEISEEDIPGEWK
S25920	A2ASS6	−0.0488367	0.044418	3	1	1	Class 1	PGPPQNLAVKEVRKDSVLLVWEPPIIDGGAK
S22804	A2ASS6	−0.0380723	0.014722	2	1	0.805129	Class 1	VIEAQRKGSDQWTHISTVKGLECVVRNLTEG
S34611	A2ASS6	−0.0345249	0	2	1	0.68296	Class2	TGQSFKSIHEQVSSISETTKSVQKTAESAEA
S30572	A2ASS6	−0.0335397	0	2	1	0.832922	Class 1	DGGTEITNYIVEKRESGTTAWQLINSSVKRT
S35063	A2ASS6	−0.0245479	0	2	1	0.632417	Class2	KQEASFSSFSSSSASSMTEMKFASMSAQSMS
S4018	A2ASS6	−0.018641	0	2	1	0.675564	Class 2	VLLKEEQSEVVAVPTSQTSKSEKEPEAIKGV
S35036	A2ASS6	−0.0161605	0	2	1	0.952755	Class 1	REVVLKTSSDVSLHGSVSSQSVQMSASKQEA
S3991	A2ASS6	−0.0149712	0.0107483	2	1	0.999502	Class 1	KSTGQDVALRTEEGKSLSFPLALEEKQVLLK
S17113	A2ASS6	−0.01187	0.0154739	3	1	0.985972	Class 1	STITLDWKEPRSDGGSPIQGYIIEKRRHDKP
S814	A2ASS6	−0.0108688	0.0162498	2	1	0.999999	Class 1	STERLVHVDKRPRTASPHFTVSKISVPKTEH
S20755	A2ASS6	−0.0093163	0.0181907	2	1	1	Class 1	DGSSVLIIKDVTRKDSGYYSLTAENSSGSDT
S27556	A2ASS6	−0.0092088	0.0073245	2	1	0.999755	Class 1	DQRYEFRVFARNAADSVSEPSESTGPITVKD
S3870	A2ASS6	−0.0051650	0.0056415	3	1	0.991661	Class 1	EPEGVFPGASSAAQVSPVTIKPLITLTAEPK
S4672	A2ASS6-3	−0.0048496	0.0087214	3	1	1	Class 1	ENGDKTFISQLKRAASEEECLEDHEMEDGPT
S28731	A2ASS6	−0.0040724	0.0043022	2	1	0.974739	Class 1	LASILIKDANRLNSGSYELKLRNAMGSASAT
S17109	A2ASS6	0.00032406	0	3	1	0.836525	Class 1	ERTKSTITLDWKEPRSDGGSPIQGYIIEKRR
S14664	A2ASS6	0.0113125	0.0110327	2	1	1	Class 1	REIKEGKKYKFEKDGSIHRLIIKDCRLEDEC
T21632	A2ASS6	0.0207359	0	2	1	0.819558	Class 1	AERKSWSTVTTECSKTSFRVSNLEEGKSYFF
S17316	A2ASS6	0.02154	0	3	1	0.980872	Class 1	ESCYLTWDAPLDNGGSEITHYIIDKRDASRK
S34623	A2ASS6	0.0307702	0.0977465	2	1	1	Class 1	SSISETTKSVQKTAESPEAKKQEPIAPESIS
S756	A2ASS6	0.0341452	0.0943439	3	1	1	Class 1	HISTTKVPEQPRRPASEPHVVPQAVKPAVIQ
S34573	A2ASS6	0.0437442	0	3	1	0.978092	Class 1	SADGTYELKIHNLSESDCGEYVCEVSGEGGT
S34571	A2ASS6	0.120101	0	2	1	0.99376	Class 1	HYSADGTYELKIHNLSESDCGEYVCEVSGEG
T32113	A2ASS6	0.130455	0	3	1	0.999962	Class 1	GISKPLKSEEPVIPKTPLNPPEPPSNPPEVL
T25315	A2ASS6	0.134102	0	3	1	1	Class 1	NSECYVARDPCDPPGTPEAIIVKRNEITLQW
S2078	A2ASS6	0.155502	0.903734	2	1	1	Class 1	GKITIPTFKPERIELSPSMEAPKIFERIQSQ
T21345	A2ASS6	0.156475	0.125556	3	1	0.999996	Class 1	PVIERTLKATGLQEGTEYEFRVTAINKAGPG
S31438	A2ASS6	0.158509	0	2	1	0.78457	Class 1	VPLVPTKLEVVDVTKSTVTLAWEKPLYDGGS
S34488	A2ASS6	0.158631	0	2	2	0.999994	Class 1	RVKSPEPVTSHPKAVSPTETKPTEKGQHLPV
S34476	A2ASS6	0.159999	0	2	2	1	Class 1	RVKSPETVKSPKRVKSPEPVTSHPKAVSPTE
S35128	A2ASS6	0.165174	0.213397	2	1	0.999984	Class 1	GRGIPPKIEALPSDISIDEGKVLTVACAFTG
S315	A2ASS6	0.166903	0.183848	2	2	0.997856	Class 1	PSPVRSVSPAGRISTSPIRSVKSPLLIRKTQ
S22797	A2ASS6	0.170233	0.274145	2	1	1	Class 1	GSKITGYVIEAQRKGSDQWTHISTVKGLECV
S25870	A2ASS6	0.199285	0	2	1	1	Class 1	TDKVQIEKGINFTQLSIDNCDRNDAGKYILK
S13764	A2ASS6	0.200173	0	2	1	1	Class 1	SWFKNDQRLHTSKRVSMHDEGKTHSITFKDL
S814	A2ASS6	0.200474	0.566341	2	2	0.999999	Class 1	STERLVHVDKRPRTASPHFTVSKISVPKTEH
S21152	A2ASS6	0.207222	0.196717	2	1	1	Class 1	RNLCTLELFSVNRKDSGDYTITAENSSGSKS
S307	A2ASS6	0.21303	0.565178	2	1	1	Class 1	VRHVRAPTPSPVRSVSPAGRISTSPIRSVKS
S2078	A2ASS6	0.216232	0.446394	2	2	1	Class 1	GKITIPTFKPERIELSPSMEAPKIFERIQSQ
S2080	A2ASS6	0.216232	0.446394	2	2	1	Class 1	ITIPTFKPERIELSPSMEAPKIFERIQSQTV
S33353	A2ASS6	0.219718	0.237812	3	1	1	Class 1	IRSQRGVSVAKVKVASIEIGPVSGQIMHAIG
T812	A2ASS6	0.223205	0.643762	4	2	0.999999	Class 1	DISTERLVHVDKRPRTASPHFTVSKISVPKT
Y33436	A2ASS6	0.229236	0	2	1	0.996224	Class 1	TKFDDGTYRCKVVNDYGEDSSYAELFVKGVR
S264	A2ASS6	0.246749	0.784633	2	1	1	Class 1	PHKTPPRIPPKPKSRSPTPPSIAAKAQLARQ
S3676	A2ASS6	0.258327	0	4	1	0.878152	Class 1	EDMPLYTSVCYTIIHSPDGSGTFIVNDPQRG
S879	A2ASS6	0.26138	0.407205	3	1	0.998042	Class 1	TVKPGETRVRAEPTPSPQFPFADMPPPDTYK
S1214	A2ASS6	0.287447	0.235319	2	1	1	Class 1	IQEPKVGEIAPGFAYSEYEKEYEKEQALIRK
S322	A2ASS6	0.325277	3.06343	2	1	1	Class 1	SPAGRISTSPIRSVKSPLLIRKTQTTTMATG
S12678	A2ASS6	0.331543	0.65471	3	1	1	Class 1	IEKPKLKPRPPARPPSPPKEDVKEKMFQLKA
T266	A2ASS6	0.332311	0.771702	2	2	1	Class 1	KTPPRIPPKPKSRSPTPPSIAAKAQLARQQS
S2032	A2ASS6	0.353204	0.75905	2	1	1	Class 1	EAITAVELKSRKKDESYEELLKKTKDELLHW

Novex-3 (A2ASS6-3).

Z-disk.

Proximal Ig region.

N2B-element.

Middle Ig region.

PEVK.

Distal Ig domains.

**A-band**.

M-band.

### Titin Isoform Composition in PKD1 cKO Mice

To study whether PKD has an influence on titin isoform composition, homogenized myocardial samples of cKO and matched WT mice were separated using SDS-PAGE, the protein bands visualized by Coomassie staining and the two cardiac titin isoforms N2BA and N2B analyzed using densitometry; a representative titin gel showing these two isoforms in WT and cKO is shown in Figure 2C. On average, titin isoform composition remained unchanged in cKO vs. WT mouse hearts. For both groups, the ratio of N2B to N2BA was ~85:15% (Figure 2B). A “T2” titin degradation band was also detectable in hearts of both cKO and WT, but showed no significant change.

### Site-Specific Phosphorylation at Titin-N2Bus and Titin-PEVK

To confirm titin phosphorylation sites identified by quantitative MS and to analyse site-specific titin phosphorylation, affinity-purified anti-phosphosite-specific antibodies were generated against conserved serines of human/mouse N2Bus at Ser4010/Ser3991, Ser4062/Ser4043, and Ser4099/Ser4080, as well as human/mouse PEVK at Ser11878/Ser12742 and Ser12022/Ser12884. The PVDF staining served as a protein loading control.

Using the titin anti-phospho-antibodies we found unaltered phosphorylation at the positions Ser3991 (Figure 2C) and Ser4080 (Figure 2E) but significantly increased phosphorylation at the positions Ser4043 (Figure 2D), Ser12742 (Figure 2F), and Ser12884 (Figure 2G) in cKO compared to WT hearts. Using an anti-phosphor-Ser/Thr antibody, overall-titin phosphorylation was found to be greatly decreased, by ≈25% on average, in cKO compared to WT hearts (Figure 2H).

### Cardiomyocyte F_Passive_ Is Elevated in cKO Hearts

**Figure 3:**
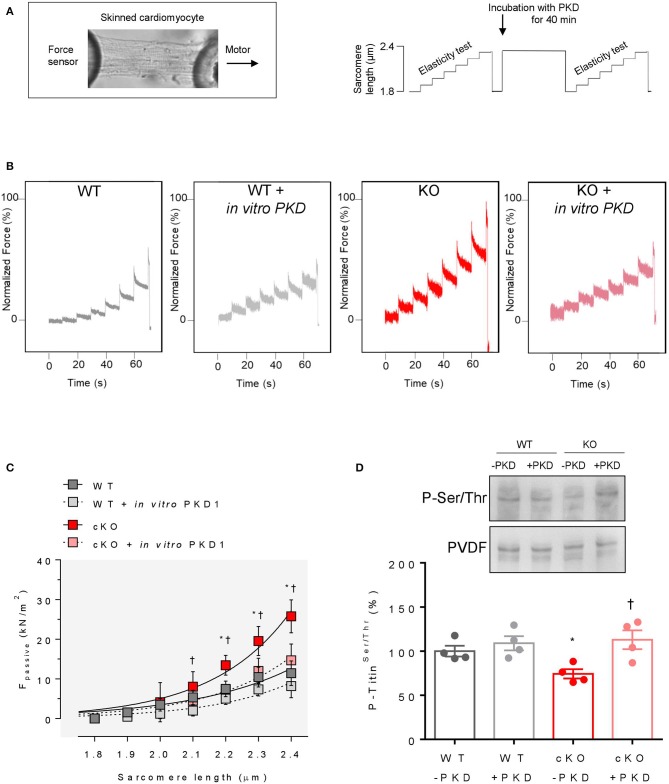
Cardiomyocyte passive stiffness and overall-titin phosphorylation in WT and PKD1 cKO mice by back-phosphorylation. **(A)** Representative image (left) and stretch protocol (right) used in the experiments **(B)** Original recordings of the force response to stepwise cell stretching of isolated skinned cardiomyocytes. **(C)** WT and PKD1 cKO F_passive_ at sarcomere length 1.8–2.4 μm in the presence or absence of *in vitro* PKD1 (16–20 cardiomyocytes/group). Curves are second-order polynomial fits to the means (± SEM; *n* = 4 cardiomyocytes/heart). For B; *P < 0.05 WT vs. cKO, ^†^*P* < 0.05 cKO vs. cKO after PKD treatment in Student *t*-test. **(D)** Representative immunoblot and graph before (–PKD1) and after incubation with protein kinase D1 (+PKD1). Data are means ± SEM; *n* = 4 hearts/group. Each heart was analyzed in duplicate. *P < 0.05 in Students unpaired and paired *t*-test.

The passive length–tension relationship of single skinned cardiomyocytes in relaxing solution was steeper in cKO than in WT hearts (Figures 3A,B). Significantly increased F_passive_ levels in cKO vs. WT cells were found at sarcomere lengths (SL) of 2.1 μm or higher. Administering PKD to non-activated skinned cardiomyocytes from cKO hearts in relaxing solution significantly reduced F_passive_ at SL 2.2 μm or higher, returning it to levels found in matched WT cells (Figure 3C), whereas administration of PKD to WT cardiomyocytes did not affect F_passive_.

In an alternative approach, demembranated cardiac fiber bundles from hearts of these mice were phosphorylated *ex vivo* by PKD and then titin SDS-PAGE was performed followed by Western blot analysis. Titin phosphorylation was significantly increased after PKD treatment in cKO hearts, while in WT it remained unaltered (Figure 3D).

### Increased PKD and HSP27 Activity Is Associated With Increased Oxidative Stress

**Figure 4:**
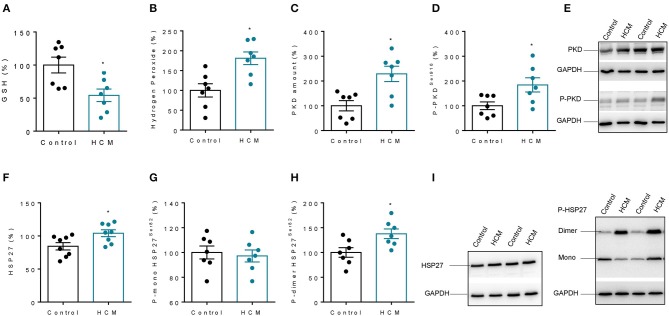
Myocardial oxidative stress parameters, PKD-content and phosphorylation, and HSP27-content and phosphorylation in HCM patients. **(A)** Reduced Glutathione (GSH). **(B)** Hydrogen Peroxide (H_2_O_2_). **(C)** Protein Kinase D (PKD). **(D)** Phospho-Ser916 PKD. **(E)** Representative blots of (Phospho)PKD and GAPDH. **(F)** Heat Shock Protein 27 (HSP27). **(G)** Phospho-monomer HSP27. **(H)** Phospho-dimer HSP27. **(I)** Representative blots of HSP27 and monomer and dimer of HSP27. Data are means ± SEM; *n* = 7–8 hearts/group.

In HCM hearts, we found less reduced glutathione (GSH) and increased hydrogen peroxide (H_2_O_2_), both of which indicate that oxidative stress was increased in HCM hearts compared to control non-failing hearts (Figures 4A,B). Myocardial PKD content and PKD phosphorylation at phosphosite Ser916 were both significantly higher in HCM hearts compared to controls, indicating total myocardial PKD activity in HCM (Figures 4C–E). In addition, the amount of HSP27 was higher in hearts of HCM patients compared to controls (Figure 4F) and the phosphorylation of HSP27 resulted in an altered dimer phosphorylation of HSP27 in HCM compared to controls, while HSP27 monomers remained unchanged (Figures 4G–I).

### Cardiomyocyte F_Passive_ Is Elevated in HCM Hearts

**Figure 5:**
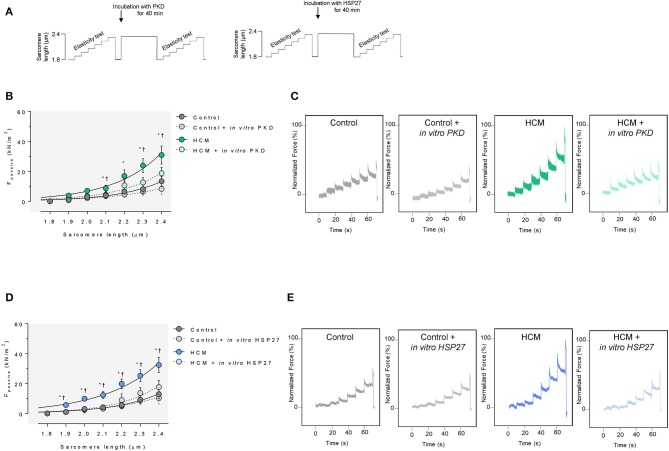
Cardiomyocyte passive stiffness in human non-failing and failing HCM hearts. **(A)** Stretch protocol used in experiments. **(B)** F_passive_ at sarcomere length 1.8–2.4 μm in the presence or absence of *in vitro* human recombinant PKD of human non-failing control and failing HCM. **(C)** Original recordings of the force response to stepwise cell stretching of isolated skinned cardiomyocytes. Curves are second-order polynomial fits to the means (± SEM; *n* = 4 cardiomyocytes/heart). For **(B)**; *P < 0.05 Control vs. HCM, ^†^*P* < 0.05 HCM vs. HCM after PKD or HSP27 treatment in Student *t*-test. **(D)** Human non-failing control and failing HCM F_passive_ at sarcomere length 1.8–2.4 μm in the presence or absence of *in vitro* human recombinant HSP27. **(E)** Original recordings of the force response to stepwise cell stretching of isolated skinned cardiomyocytes.

Cardiomyocytes from human end-stage failing hearts (only from patients with hypertrophic cardiomyopathy) showed a higher cardiomyocyte F_passive_ at sarcomere length 2.1 μm or higher compared to non-failing human heart samples. Administering human recombinant PKD to non-activated skinned cardiomyocytes from human HCM hearts in relaxing solution significantly reduced F_passive_ at sarcomere length 2.1 μm or higher, lowering it to levels found in cells from control hearts (Figures 5A–C). In addition, upon treatment with human recombinant HSP27, F_passive_ was significantly reduced at all sarcomere lengths, whereas control cardiomyocytes remained unaltered (Figures 5D,E).

### Titin Phosphorylation in Hearts of Human HCM Patients

**Figure 6:**
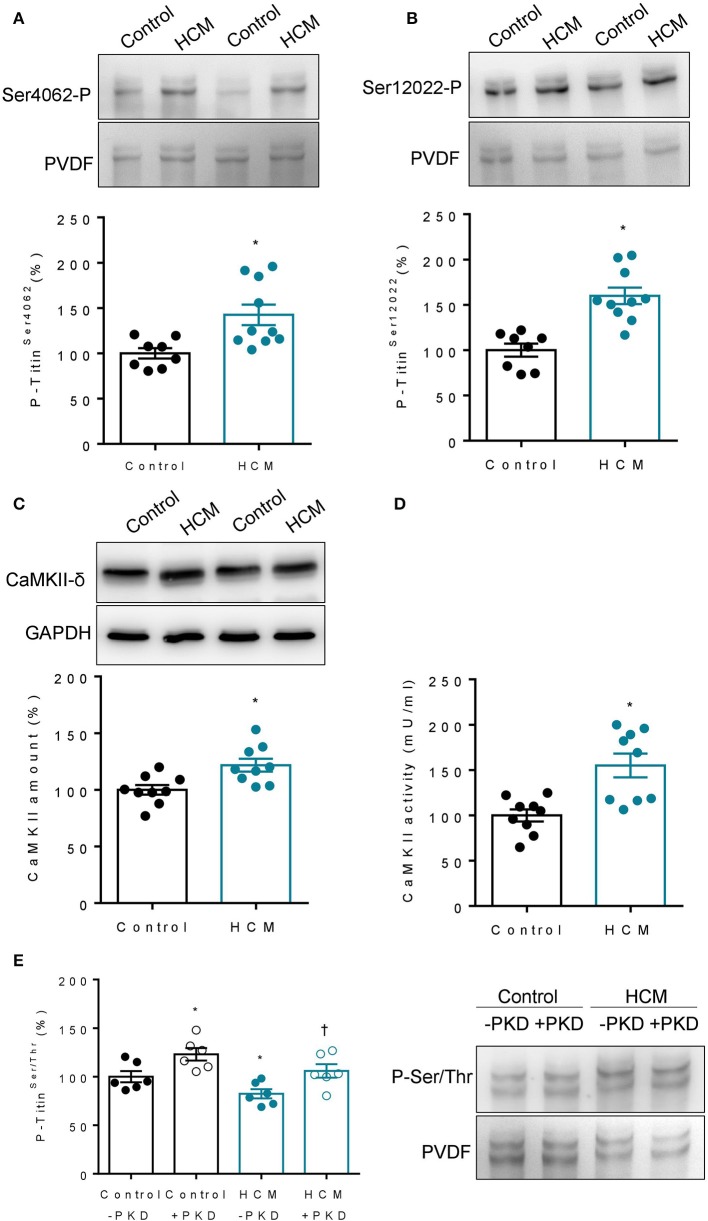
Titin phosphorylation and CaMKII content and activity in hearts of failing HCM and non-failing control patients. **(A)** Site-specific phosphorylation at position Ser4062 in N2Bus (*n* = 8 − 10 hearts/group; each heart analysed in duplicate). **(B)** Site specific phosphorylation at position Ser12022 in PEVK region (*n* = 8 − 10 hearts/group; each heart analysed in duplicate). **(C)** CaMKII content in HCM vs. control hearts (*n* = 9 hearts/group). or absence of *in vitro* PKD. **(D)** CaMKII activity in HCM vs. control hearts (*n* = 9 hearts/group). **(E)** Total N2B-titin phosphorylation in HCM vs. control hearts (*n* = 8 − 10 hearts/group; each heart analysed in duplicate). Representative immunoblot right panel and graph left panel before (–PKD) and after incubation with protein kinase D1 (+PKD). Data are means ± SEM; *n* = 6 hearts/group. Each heart was analyzed in duplicate. *P < 0.05 vs. Control untreated; ^†^*P* < 0.05 vs. HCM untreated.

We found increased CaMKII phosphosites at titin positions Ser4062 and Ser12022 in HCM compared to control hearts (Figures 6A,B), which went along with increased myocardial CaMKII content and activity (Figures 6C,D). Total titin phosphorylation was significantly lower in HCM compared to non-failing hearts (Figure 6E). Using an alternative approach, we measured total titin phosphorylation in demembranated cardiac fiber bundles obtained from HCM and non-failing human hearts before and after *ex vivo* phosphorylation by human recombinant PKD (Figure 6E). Strong signals were seen for both isoforms N2BA and N2B after kinase treatment, and the total phosphorylation of titin was significantly increased by more than 20% in non-failing and failing HCM hearts.

### Subcellular Localization and Overlay of PKD and HSP27 in HCM and Control Hearts

**Figure 7:**
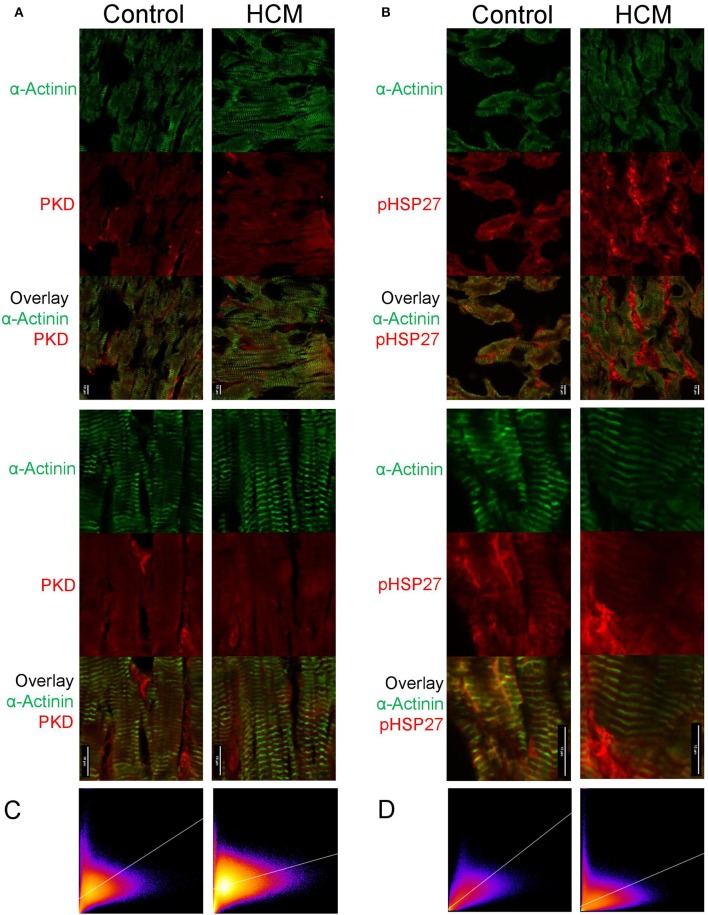
Images of confocal laser scanning microscopy of PKD or HSP27 in human cardiomyocytes from failing HCM and control hearts. **(A)** Representative immunohistochemistry of PKD labeling in non-failing control human biopsy specimens along with a volume view. **(B)** Representative immunohistochemistry of PKD labeling in HCM human biopsy specimens along with a volume view. **(C)** 2D intensity histograms showing the relationship of intensities at the exact position between two channels representing alpha-actinin and PKD. **(D)** 2D intensity histograms showing the relationship of intensities at the exact position between two channels representing HSP-27 and alpha-actinin.

**Figure 8:**
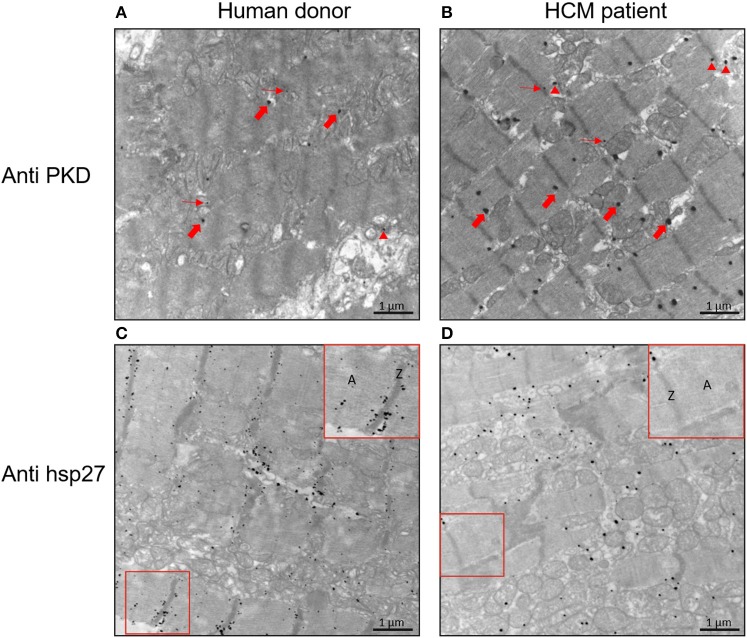
Electron microscopy images to illustrate sarcomeric localization of PKD and HSP27 in human cardiomyocytes of failing HCM and non-failing control hearts. **(A)** Representative electron microscopy images with PKD labeling in non-failing control human. **(B)** Representative electron microscopy images with PKD labeling in HCM. **(C)** Representative electron microscopy images with HSP27 labeling in non-failing control human. **(D)** Representative electron microscopy images with HSP27 labeling in HCM. Small red arrow is for monomer; arrowhead red is for dimer; and big red arrow is for polymer.

As we found a relatively high level of PKD expression in failing HCM hearts using western blot, we wished to determine the cellular distribution of PKD using confocal laser scanning and electron microscopy. The confocal images showed a clear overlay of PKD signals with sarcomeres in control and HCM hearts (Figure 7A). Phospho-HSP27 was clearly present at the periphery of the cardiomyocytes in HCM compared to control hearts, whereas in control hearts, phospho-HSP27 was distributed throughout the cardiomyocyte (Figure 7B). 2D intensity histograms show two channels and the level of overlapping. For a high overlapping level, the histogram pixels tend to concentrate more along the y = x line. 2D intensity histogram analysis showed a more intense presence of PKD in HCM but distribution was not significantly different, while phospho-HSP27 in HCM hearts compared to controls showed less correlation with α-actinin as more pixels concentrate at the axis of the histogram, the less correlation can be expected (Figures 7C,D). In keeping with these findings, electron microscopy confirmed the higher level of PKD in myocardium HCM compared to control hearts (Figures 8A,B). Using electron microscopy, HSP27 showed a strongly altered localization away from the Z-disk and A-band in HCM, whereas HSP27 was located preferentially at the Z-disk and A-band in controls (Figures 8C,D).

## Discussion

**Figure 9:**
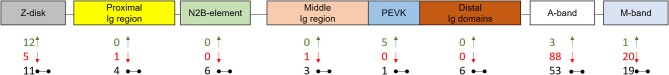
Overview of identified phosphosites on the entire titin molecule Figure shows all detected phosphosites on the Z-disk, Proximal Ig region, N2B-element, Middle Ig region, PEVK, Distal Ig domains, A-band, and M-band. 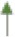
Upregulated, 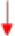
downregulated, and 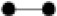 unchanged.

PKD has emerged as a key regulator of excitation-contraction coupling and cardiac hypertrophic signaling. Cardiac-specific PKD1-cKO mice show resistance to cardiac hypertrophy and fibrosis in response to pressure overload and angiotensin II treatment (Fielitz et al., 2008). Overexpression of constitutively active PKD1 in mouse hearts leads to dilated cardiomyopathy, and an increase in PKD1 expression and activity is seen in failing hearts of rats (Harrison et al., 2006), rabbits and humans (Bossuyt et al., 2008). These findings indicate the importance of PKD1 in regulating cardiac pathophysiology and the potential of the kinase as a therapeutic target in cardiovascular disease (Nichols et al., 2014). As PKD1 showed some beneficial effects on cardiomyocyte function (Haworth et al., 2004; Cuello et al., 2007), we wanted to know if PKD is also involved in the phosphorylation of titin. We demonstrated that titin is an important substrate of PKD. In a quantitative MS screen using PKD1-cKO hearts and the SILAC mouse heart, we found that titin was among the cardiac proteins most highly affected by PKD1 deletion. Overall, titin phosphorylation was significantly reduced in cKO compared to WT hearts. Many of the phosphosites found in titin are located within the Z-disk, A-band, or M-band sections of the molecule, where they could be involved in regulating protein–protein interactions or mechanical signaling (Figure 9). In addition, we identified many phosphosites within the I-band titin region (proximal Ig region, N2-Bus and PEVK region) present in the N2BA and N2B cardiac titin isoforms (Figure 9). Furthermore, we showed that PKD reduces cardiomyocyte stiffness in PKD1 cKO mice and in failing hearts of human HCM patients. As PKD1 activation and increased PKD1 phosphorylation is observed in the hypertrophic heart from mice that have undergone tac surgery and as PKD plays an important role in the development of cardiac hypertrophy as shown via inhibition of hypertrophy in isolated neonatal cardiomyocytes from Wistar rats with PKD deletion (Zhao et al., 2017, 2019), we used human HCM tissues to validate how important is PKD in human heart failure with hypertrophy. We found disturbed titin phosphorylation in human HCM hearts, possibly contributing to the mechanical dysfunction. This was associated with increased PKD and CaMKII content and activity in these hearts. Moreover, we detected an increased amount and phosphorylation of HSP27, a substrate of PKD, in human HCM hearts. Changes in PKD, CaMKII and HSP27 were associated with increased oxidative stress parameters. Our work reveals a previously unknown role of PKD in regulating diastolic passive properties of healthy and diseased hearts, and its association with oxidative stress and changes affecting HSP27.

### Titin Is a Substrate of PKD

Changes in titin stiffness due to isoform switching or post-translational modifications such as phosphorylation and oxidation have been reported in a variety of species. Previous studies showed that heart failure is associated with a chronic deficit in global titin phosphorylation (Bishu et al., 2011; Falcao-Pires et al., 2011; Hamdani et al., 2013a,b). Titin is phosphorylated by PKA at the N2B spring element, which results in a reduction of cardiomyocyte F_passive_ (Yamasaki et al., 2002; Kruger and Linke, 2006). In addition, titin is phosphorylated by PKG at the N2Bus (Kruger et al., 2009; Kotter et al., 2013), a modification that also reduces cardiomyocyte F_passive_ (Borbely et al., 2009; Hamdani et al., 2013a, 2014). Following short-term cGMP-enhancing treatment with sildenafil and B-type natriuretic peptide (BNP) in an animal model of heart failure with preserved ejection fraction (HFpEF) (elderly hypertensive dogs) (Bishu et al., 2011), PKG-mediated phosphorylation of titin resulted in acutely increased cardiac extensibility and may also positively regulate hypertrophic signaling. Our study provides the first evidence that PKD also phosphorylates titin. Notably, altered phosphorylation of titin sites was seen within the PEVK-domain, especially at the phosphosites that are also CaMKII-dependent. Perhaps this hints at a compensatory mechanism due to the loss of PKD in these mice.

### Cardiomyocyte Stiffness and PKD

Cardiomyocyte F_passive_ was elevated in cKO compared to WT, and incubating skinned cardiomyocytes with PKD lowered F_passive_ in cKO, but did not affect WTs. Furthermore, cardiomyocyte isolated from failing HCM hearts showed increased F_passive_, which could be reduced upon treatment with PKD enzyme. Thus, PKD alters F_passive_ in the same direction as PKA, PKG, ERK2, and CaMKIIδ, an effect that may depend mainly on the phosphorylation of N2Bus. The latter increases the persistence length of N2Bus and thereby reduces F_passive_ (Kruger et al., 2009). Another possible mechanism is via phosphorylation of titin Ig domains, although this is not yet proven. To date, PKCα is the only kinase that increases cardiomyocyte stiffness via phosphorylation of the PEVK region. The mechanical effect of PKCα-mediated PEVK phosphorylation includes increased persistence length of PEVK causing increased stretch-dependent force of the titin spring, thus elevating cardiomyocyte passive tension (Hidalgo et al., 2009). Reduced all-titin phosphorylation contribute to the development of heart failure, against the reduced phosphorylation, increased phosphorylation has been demonstrated in heart failure, the PKCα-dependent phosphosite at S11878 within the PEVK-titin segment was hyperphosphorylated in HFpEF animal models (Hamdani et al., 2013a, 2014; Linke and Hamdani, 2014; Franssen et al., 2016). PKCα was shown to be increased in heart failure (Belin et al., 2007) and in the HFpEF dog hearts. We believe what might determine the spatial arrangement phosphorylation along the titin molecule is the distinct micro-environment of phospho-sites either surrounded by negatively charged micro-environment and phospho-sites located at hydrophobic environment and close to cysteines sites. This may induce distinct biological and mechanical responses that may have differential effects on heart muscle exposed to oxidative stress and inflammation.

In failing human hearts, the activities of PKA and PKG are typically reduced (Hamdani et al., 2013a, 2014; Linke and Hamdani, 2014; Franssen et al., 2016), whereas the activity and the expression of PKCα, ERK2, and CaMKIIδ are usually increased, perhaps because of the presence of hypertrophy. In human failing HCM hearts, cardiomyocyte F_passive_ was increased compared to non-failing hearts, an effect that was related to increased CaMKII activity and consequently, increased phosphorylation of titin within the PEVK region at CaMKIIδ-dependent phosphosites (Bishu et al., 2011; Falcao-Pires et al., 2011; Hamdani et al., 2013a,b). Cardiomyocyte F_passive_ has been found to be pathologically elevated in human HFpEF and heart failure with reduced ejection fraction (HFrEF), due to disturbed activity of PKA and PKG. While inhibition of PKCα activity has been suggested as a potential therapeutic target for hypertrophied hearts, boosting PKD enzyme activity in failing, overly stiff, hearts could be beneficial for cardiomyocyte stiffness, similar to PKA, PKG, and ERK2 (Borbely et al., 2009; Raskin et al., 2012; van Heerebeek et al., 2012; Hamdani et al., 2013a, 2014; Kotter et al., 2013; Linke and Hamdani, 2014). A reduction in PKD-mediated titin phosphorylation, in turn, would increase F_passive_ and be detrimental to diastolic filling. However, a stiffer titin spring could speed up diastolic recoil and amplify some of the mechanosensory functions of titin.

### The PKD Substrate HSP27

The current study also showed that HSP27 content and phosphorylation is increased in human HCM hearts, a mechanism that might work to protect cardiomyocytes from damage, as an established function of HSP27 is to prevent stress-induced protein aggregation and myocardial damage (Linke and Hamdani, 2014). The observed increase in HSP27 was associated with increased oxidative stress and increased content and activity of PKD, which also phosphorylates HSP27. PKD showed a polymer formation on EM, which indicates that PKD is oxidized. This may thus explain the beneficial *ex-vivo* effect of PKD on cardiomyocyte F_passive_ and indicates that perhaps increased PKD activity observed in HCM hearts is partially due to PKD oxidation as well-suggesting that by lowering oxidation we may improve cardiomyocyte function in a manner similar to administration of endogenous PKD *ex vivo*. PKD is an important downstream regulator of the oxidative stress response, and increased oxidative stress leads to the activation of different PKD isoforms and subsequent phosphorylation of HSP27 (Doppler et al., 2005; Stetler et al., 2012). In our study, phosphorylation of HSP27 was increased in HCM hearts, which is in line with previous findings showing that HSP27 phosphorylation prevents apoptosis by protecting cells against heat shock, apoptosis effectors, oxidative stress, and ischemia (Martin et al., 1997; Benjamin and McMillan, 1998; Knowlton et al., 1998; Yoshida et al., 1999; Dohke et al., 2006; Li et al., 2012). HSP27 is abundant in cardiac muscle cells, commonly localized to the Z-disk and I-band regions of the sarcomere, and involved in the protection of titin and intermediate filaments (Kotter et al., 2014; Linke and Hamdani, 2014). In HCM hearts, HSP27 showed a strongly altered localization away from the Z-disk and I-band, whereas in non-failing hearts, HSP27 was preferentially localized to the Z-disk and I-band. Whether the translocation of HSP27 depends on the altered phosphorylation status of HSP27 is still controversial (Mymrikov et al., 2011); altered phosphorylation of HSP27 at least did not alter the binding to titin domains (Kotter et al., 2014; Linke and Hamdani, 2014). HSP27 can function as an inhibitor of cell death upon its phosphorylation and dissociation to lower-molecular-weight oligomers, while the presence of the high-molecular-weight, non-phosphorylated form of HSP27 appears to be necessary for cellular protection against cardiac ischemia/reperfusion (Golenhofen et al., 2002, 2006; Kadono et al., 2006; Pinz et al., 2008). PKD specifically phosphorylates HSP27 in pancreatic cancer cells (Yuan and Rozengurt, 2008) and endothelial cells (Evans et al., 2008). Phosphorylation of Ser82 is considered to be the main “effector” step for the shift from large molecular weight multimers to differentially functional oligomers. As regards titin, HSP27 specifically protects the unfolded Ig regions, but not the intrinsically disordered segments (N2Bus and PEVK), from aggregating under acidic stress (Kotter et al., 2014). The protective role of small HSPs on titin extensibilitywas also evident in earlier studies in which α-B crystallin lowered the persistence length of the N2Bus segment and reduced the unfolding probability of the immunoglobulin domains flanking the N2Bus segment (Bullard et al., 2004). Isolated hearts lacking sHSPs (DKO) showed severe contractile dysfunction (Pinz et al., 2008), increased myocardial injury and resting tension (Golenhofen et al., 2006), accompanied by diastolic dysfunction in response to ischemia/reperfusion *ex vivo* (Pinz et al., 2008). This accords with our previous findings in isolated human cardiomyocytes in which under conditions promoting titin aggregation (pre-stretch and acidic pH), passive stiffness was high in the absence of sHSPs but normal in the presence of sHSPs (Kotter et al., 2014). In our present work on human HCM cardiomyocytes, HSP27 lowered F_passive_ to the levels previously reported after administration of PKA and/or PKG (Borbely et al., 2005; Fukuda et al., 2005; Kruger et al., 2009; Falcao-Pires et al., 2011). In addition, F_passive_ fell to the level observed after PKD administration.

## Conclusion

Our findings have important therapeutic implications as they imply that drugs that balance PKD activity and restore HSP27 localization to the Z-disk and I band may show efficacy as a treatment for diastolic LV dysfunction related to high cardiomyocyte stiffness.

## Data Availability Statement

All datasets generated for this study are included in the article/Supplementary Material.

## Ethics Statement

All animal procedures were performed in accordance with the guidelines of Charité Universitätsmedizin Berlin as well as Max-Delbrück Center for Molecular Medicine and were approved by the Landesamt für Gesundheit und Soziales (LaGeSo, Berlin, Germany) for the use of laboratory animals (permit number: G 0229/11) and followed the Principles of Laboratory Animal Care (NIH publication no. 86-23, revised 1985) as well as the current version of German Law on the Protection of Animals. The studies involving human participants were reviewed and approved by (St Vincent's Hospital of Sydney, Australia, Human Research Ethics Committee; File number: H03/118; Title: Molecular Analysis of Human Heart Failure. The patients/participants provided their written informed consent to participate in this study.

## Author Contributions

MH has generated all mass spectrometry and biochemistry data, analyzed all data, and has written the manuscript. DK has generated the confocal images and written the manuscript. ML has made the electron microscopy. SH and MK helped and supervised the mass spectrometry. ÁK performed mechanic experiments. ZP contributed to mechanics and rewrote the manuscript. KJ helped with re-analyzing the biochemistry data and rewrote the manuscript. PH provided the tissues. CD provided the human HCM biopsies. PR re-analyzed some data. AM re-analyzed the data and rewrote the manuscript. JF provided the Prkd1 mice and rewrote the manuscript. WL supervised and rewrote the manuscript. NH supervised, re-analyzed all data, performed mechanics, and wrote the manuscript.

### Conflict of Interest

SH was employed by the company Sanofi-Aventis Deutschland GmbH Industriepark Höchst. The remaining authors declare that the research was conducted in the absence of any commercial or financial relationships that could be construed as a potential conflict of interest.
