# Supplementary material for: Modulation of Titin-Based Stiffness in Hypertrophic Cardiomyopathy via Protein Kinase D
Source: Front Physiol. 2020 Apr 15;11:240. doi: 10.3389/fphys.2020.00240 (PMC7174613; doi:10.3389/fphys.2020.00240)
Supplement: Supplementary file 3 [file Data_Sheet_1.pdf]

SUPPLEMENTARY METHODS

Human heart tissues

LV tissue was obtained during heart transplantation surgery from end-stage heart failure patients (NYHA class III or IV; n=10), hypertrophic cardiomyopathy (all male and average age, 45 years). All hearts presented with hypertrophic cardiomyopathy and obtained during cardiac transplantation surgery. Medication included angiotensin converting enzyme inhibitors, angiotensin-II-receptor, β-blockers, digoxin, or anti-arrhythmic agents. LV tissue from non-failing donor hearts (n=10; male; average age, 40 years) served as reference, non-failing cardiac LV tissue was obtained from donor hearts (n = 5) for which no suitable transplant recipient was found. The donors had no history of cardiac disease, a normal ECG and normal ventricular function on echocardiography performed within 24 h prior to heart explantation. Tissues were collected in cardioplegic solution and stored in liquid nitrogen until use. Samples were obtained after informed consent and with approval of the local Ethics Committee (St Vincent's Hospital of Sydney, Australia, Human Research Ethics Committee; File number: H03/118; Title: Molecular Analysis of Human Heart Failure). The investigation conforms to the principles outlined in the Declaration of Helsinki.

Cardiomyocyte specific *Prkd1* knock-out mice

All animal procedures were performed in accordance with the guidelines of Charité Universitätsmedizin Berlin as well as Max-Delbrück Center for Molecular Medicine and were approved by the Landesamt für Gesundheit und Soziales (LaGeSo, Berlin, Germany) for the use of laboratory animals (permit number: G 0229/11) and followed the 'Principles of Laboratory Animal Care' (NIH publication no. 86-23, revised 1985) as well as the current version of German Law on the Protection of Animals. The generation and usage of the conditional *Prkd1* allele was published elsewhere (Fielitz et al., 2008;Kim et al., 2008). The Cre-loxP recombination system was used for the generation of a conditional *Prkd1* allele. *Prkd1*loxP/loxP mice were crossed with Cre carrying mice controlled by cardiomyocyte-specific α-myosin-heavy-chain promotor (αMHC-Cre) (Agah et al., 1997) (cKO, *Prkd1*loxP/loxP;

αMHC-Cre). αMHC-Cre-negative littermates were used as controls (WT, *Prkd1*loxP/loxP). Cardiac tissue was obtained when mice were 8-10 weeks of age. N=7 for both KO and WT.

SILAC mouse

For the identification of PKD1-dependent phosphorylation sites, quantitative MS in combination with a modified version of the SILAC technique was used (Zanivan et al., 2012). Generally, in the SILAC technique, proteins are labeled by metabolic incorporation of stable, non-radioactive amino acid isotopes. These proteins can then be used for relative quantification. This method, initially limited to cell culture, has been expanded in recent years to more complex model organisms such as zebrafish and mouse. For breeding of the SILAC mouse, a generation of the mouse strain C57BL/6 was fed with 13C6-lysine (heavy)-containing food. This led to the complete replacement of the natural isotope 12C6-lysine (light). After complete labeling this SILAC mouse can be mixed as a heavy "spike-in" standard with unlabeled samples in a ratio of 1:1 and processed for MS experiments. The ratios of the signal intensities of peptides in the mass spectrum correlated with the ratios of the peptides from the sample being compared. Thus, it was possible to compare samples from various conditions, such as WT and KO, quantitatively (Zanivan et al., 2012;Konzer et al., 2013).

SILAC-based quantitative mass spectrometry

Isolated heart tissues were homogenized in SDS lysis buffer (4% SDS in 100 mM Tris/HCl pH 7.6) using an Ultra-Turrax (Ika). For complete lysis, samples were shortly heated at 95°C and sonicated. Next, lysates were clarified by centrifugation at 16,000 g for 5 min and protein concentration was estimated using the DC protein assay (Biorad). Then we mixed equal amounts of 13Lys6 labeled heart tissue extracts from the SILAC mouse (Kruger et al., 2008) with non-labeled mouse wildtype or non-labeled mouse PKD cKO heart tissue extracts.

In-gel digestion for protein expression levels

After lysis equal amounts of 13Lys6 labeled heart tissue extracts from the SILAC mouse (Kruger et al., 2008) were mixed with non-labeled mouse wildtype or non-labeled mouse PKD

cKO heart tissue extracts. 20 µg of the proteins were separated by SDS-PAGE (4-12% NuPage Gel, Invitrogen). The gel was stained was Coomassie (Invitrogen) and the gel bands were cut into 10 equal slices per lane and digested with Lys-C (Wako), as previously described (Shevchenko et al., 2006).

In brief, the gel pieces have been washed several times with ABC buffer (50 mM ammonium bicarbonate, 50% ethanol) and then dehydrated with ethanol and rehydrated using 50 mM ammonium bicarbonate. The proteins were reduced with 10 mM DTT and alkylated. For digestion of the proteins, 40 µL Lys-C at a concentration of 12.5 ng/µL was added to each sample. Digestion was performed overnight at 37°C. Thereafter, the peptides were extracted with three different steps containing different amounts of acetonitrile and pooled together. Then, samples were concentrated in a vacuum centrifuge and desalted using C-18 stage tips made in-house (Rappsilber et al., 2007).

Enrichment of phosphopeptides using titanium dioxide beads

Phosphopeptide analysis was done as described (Olsen et al., 2006). In brief, equal amounts of 13Lys6 labeled heart tissue extracts from the SILAC mouse (Kruger et al., 2008) were mixed with non-labeled mouse wildtype or non-labeled mouse PKD cKO heart tissue extracts (7.5 mg per condition). Protein digestion was performed with the filter aided sample preparation method (Wisniewski et al., 2009). Briefly, proteins were loaded on a filter unit for exchange of buffers, reduction/alkylation, and digested with the endopeptidase LysC (Wako) overnight. Acetonitrile was added to each sample at a final concentration of 30% and pH was adjusted with trifluoric acid to 2.7. Peptide solution was loaded onto a 1 mL Resource S column (GE Healthcare) connected to an Äkta purifier chromatography system (GE Healthcare). Peptides bound to the column were eluted by a linear gradient of salt-containing solvent. Following SCX chromatography, ten fractions and the flow-through were collected and subjected to phosphopeptide enrichment with titanium dioxide beads as described (Zanivan et al., 2008).

LC-MS Analysis

Following protein digestion with LysC and phosphopeptide enrichment, the ratio of

labeled:unlabeled peptides was determined by liquid chromatography (LC) and tandem mass spectrometry (Kruger et al., 2008) and used to identify the KO:WT ratio of titin phosphopeptides, as described (Drexler et al., 2012;Lundby et al., 2012). Mass spectrometric experiments were performed on a nano-flow HPLC system (Proxeon) connected to an LTQ-orbitrap Velos instrument (Thermo Fisher Scientific) equipped with a nanoelectrospray source (Proxeon), as described (Drexler et al., 2012)

Chromatographic separation was performed with in-house packed fused silica emitter with an inner diameter of 75 µm. Columns were packed with C18-AQ RepoSil-Pur (3 µm, Dr. Maisch GmbH). Peptide separation was performed with a linear gradient of 5-30% acetonitrile with 0.5% acetic acid for 150 min at a flow rate of 200 nL/min. After eluting from C18 column, peptides were ionized by electrospray ionization and transferred to the mass spectrometer.

The mass spectrometer was operated to monitor MS and MS/MS spectra. Survey full-scan MS spectra (from m/z 300–2000) were acquired in the orbitrap with a resolution of R=60,000 at m/z 400 after accumulation of 1,000,000 ions. The 15 most intense ions from the preview survey scan delivered by the orbitrap were sequenced by collision-induced dissociation in the LTQ. To improve peptide fragmentation multistage activation was applied. Mass spectra were analysed using Xcalibur, MaxQuant (Version 1.0.14.10), and automated Mascot database searching (Matrix Science Version 2.2). All tandem mass spectra were searched against the mouse International Protein Index protein sequence database (IPI, version 3.54) and concatenated with reversed copies of all sequences. The required false positive rate was set to 1% at the protein level, and maximum allowed mass deviation was set to 5 ppm in MS mode and 0.5 Da for MS/MS peaks. Cysteine carbamidomethylation was searched as a fixed modification and N-acetyl protein, oxidized methionine and phosphorylated STY as variable modifications. A maximum of 3 missed cleavages was allowed. Phosphorylation sites were considered as “class I” if localization probability was at least 0.75 (75%) and the localization score difference higher than 5 (Olsen et al., 2006).

Titin and phospho-titin analysis by Western blot (WB)

Polyacrylamide gel electrophoresis (PAGE) was performed to separate titin as previously described (Hamdani et al., 2013b). Briefly, LV tissue samples (n=7-10/samples) were solubilized in 50 mM Tris-SDS buffer (pH 6.8) containing 8 µg/mL leupeptin and 10 µL/mL phosphatase inhibitor cocktail (catalogue #P2850; Sigma-Aldrich) or in modified Laemmli buffer (50 mM Tris-HCl, pH 6.8, 8 M urea, 2 M thiourea, 3% SDS (w/v), 0.03 % ServaBlue (w/v), 10% (v/v) glycerol, 75 mM DTT). Samples were heated for 3 min at 96°C and centrifuged. Samples were applied in duplicates at concentrations that were within the linear range of the detection system (15 µg and 20 µg dry weight; checked by spectroscopic methods) and separated by agarose-strengthened 1.8% SDS-PAGE. Gels were run at 4 mA constant current for 16 h. Thereafter WB was performed to measure site-specific and total phosphorylation of titin. Following SDS-PAGE, proteins were transferred to polyvinylidene difluoride (PVDF) membranes (Immobilon-P 0.45 µm; Merck Millipore, Burlington, MA, USA). Blots were pre-incubated with 3% bovine serum albumin in Tween Tris-buffered saline (TTBS; containing: 10 mM Tris-HCl; pH 7.6; 75 mM NaCl; 0.1% Tween; all from Sigma-Aldrich) for 1 h at room temperature. Then, blots were incubated overnight at 4°C with the primary antibodies.

Anti-phospho serine (Ser)/threonine (Thr) antibody (ECM Biosciences LLC, Versailles, KY, USA; dilution 1:500) was used to assess total titin phosphorylation. Phosphosite-specific anti-titin antibodies were custom-made by Eurogentec (Seraing, Belgium) with positions in N2Bus (N2B unique sequence) and PEVK (rich in proline, glutamate, valine and lysine amino acids) domains of mouse (Mus musculus) titin according to UniProtKB identifier A2ASS6. The following rabbit polyclonal affinity purified antibodies were used:

- anti-phospho-N2Bus (Ser3991) against EEGKS(PO3H2)LSFPLA (dilution 1:500);

- anti-phospho-N2Bus (Ser4043) against QELLS(PO3H2)KETLFP (dilution 1:100);

- anti-phospho-N2Bus (Ser4080) against LFS(PO3H2)EWLRNI (dilution 1:500);

- anti-phospho-PEVK (Ser12742) against EVVLKS(PO3H2)VLRK (dilution 1:100);

- anti-phospho-PEVK (Ser12884) against KLRPGS(PO3H2)GGEKPP (dilution 1:500).

The amino acid sequences of mouse titin at Ser3991, Ser4043, Ser4080, Ser12742 and Ser12884 are identical to the amino acid sequences of mouse, and refer to human titin at Ser4010, Ser4062, Ser4099, Ser11878 and Ser12022, respectively.

After washing with TTBS, primary antibody binding was visualized using secondary horseradish peroxidase-labelled, goat anti-rabbit antibody (DakoCytomation, Glostrup, Denmark; dilution 1:10,000) and enhanced chemiluminescence (ECL Western blotting detection; Amersham Biosciences). WB signals were visualized using the LAS-4000 Image Reader and analysed with Multi Gauge V3.2 software (both from FUJIFILM Corp, Minato, Tokyo, Japan). Coomassie-based PVDF stains were saved for comparison of protein load. Finally, signals obtained from phospho-specific antibodies were normalized to signals obtained from PVDF stains referring to the entire protein amount transferred. Alternatively, all-titin phosphorylation was measured by PKD-mediated back-phosphorylation as described before (Hamdani et al., 2013a). Briefly, skinned fibers from WT and cKO mouse hearts and human controls and HCM hearts were phosphorylated ex vivo by PKD (1 µg/mL; Sigma-Aldrich) with kinase buffer 1 (abcam) for 60 min at 37°C in relaxing solution supplemented with phosphatase inhibitor cocktail (Sigma-Aldrich). The fibers were denatured, dissolved, and titin bands visualized on 1.8% SDSPAGE after incubating with the Anti-phospho serine (Ser)/threonine (Thr) ECM Biosciences LLC, Versailles, KY, USA; dilution 1:500) was used to assess total titin phosphorylation for mouse and human hearts, and furthermore, phosphosite-specific anti-titin antibodies recognizing the S4062 and S12022 were used for human hearts. Titin phosphorylation of the sum of N2BA and N2B titin were assessed as described above and signals on blots were analysed using Multi Gauge V3.2 software.

Force measurements on isolated cardiomyocytes

Force measurements were performed on single demembranated cardiomyocytes (n=12-42/5-6 heart/group) as described before. Briefly, LV samples were de-frozen in relaxing solution (containing in mM: 1.0 free Mg2+; 100 KCl; 2.0 EGTA; 4.0 Mg-ATP; 10 imidazole; pH 7.0), mechanically disrupted and incubated for 5 min in relaxing solution supplemented with 0.5%

Triton X-100 (all from Sigma-Aldrich). The cell suspension was washed 5 times in relaxing solution. Single cardiomyocytes were selected under an inverted microscope (Zeiss Axiovert 135, 40x objective; Carl Zeiss AG Corp, Oberkochen, Germany) and attached with silicone adhesive between a force transducer and a high-speed length controller (piezoelectric motor) as part of a "Permeabilized Myocyte Test System" (1600A; with force transducer 403A; Aurora Scientific, Aurora, Ontario, Canada). Cardiomyocyte Fpassive was measured in relaxing buffer at room temperature within a sarcomere-length range between 1.8 and 2.4 µm (Untreated phase), subsequently cardiomyocytes were incubated in relaxing solution supplemented with PKD (1 µg/mL; Sigma-Aldrich) and/or HSP27 (1 mg/ml; abcam) (treatment phase) for 40 min. Thereafter Fpassive measurements were again performed in relaxing solution at SL 1.8-2.4 µm. After 40-min-long incubation, Fpassive measurements were again performed in relaxing solution (SL 1.8-2.4 µm). All force values were normalized to myocyte cross-sectional area calculated from the diameter of the cells, assuming a circular shape. All the measurements were controlled as we test the cell viability, so each cardiomyocyte was also transferred from relaxing to maximally activating solution (pCa4.5), at which isometric force developed. Once a steady state force was reached, the cell was shortened within 1 ms to 80% of its original length to determine baseline force. The maximal tension was measured at the begin before baseline Fpassive and at the end of the measurements (after Fpassive measurements and PKD incubations). Cardiomyocytes with maximal tension of ±20% variation in cross-sectional area from the start to the end of experimental protocol (the maximal tension differences) were excluded from analysis, as we considered that cell damaged during the incubation time.

We followed the same protocol for human LV samples from HCM and donor hearts before and after incubations with PKD and/or HSP27. Cardiomyocyte (n=13/group) Fpassive was thereafter measured within a SL range between 1.8 and 2.4 µm as described above.

Quantification of tissue oxidative stress

Myocardial levels (n=7 LV sample/group) of oxidative stress markers were tested with enzyme-linked immunosorbent assay (ELISA). Hydrogen peroxide (H2O2) was assessed in LV tissue

homogenates (n=4-10/group). Samples containing equal amounts of total protein were analysed for H2O2 formation. H2O2 formation was measured by colorimetry at 540 nm. Results were converted using the standard curve for a known concentration of H2O2. Additional, total glutathione in plasma samples were determined in duplicate with a colorimetric glutathione assay kit (CS0260, Sigma Aldrich) to assess antioxidant levels.

Content and phosphorylation of PKD and HSP27

PKD and HSP 27 content and phosphorylation was determined using WB. The following antibodies have been used:

- Anti-Phospho-HSP27 (Ser 82) Cell Signaling Technology (#2401) (1:1000 in WB)
- Anti-HSP27 (abcam) (ab2790) (1:1000 in WB)
- Anti-Phospho-PKD/PKCμ (Ser916) Cell Signaling Technology (#2051) (1:750 in WB)
- Anti-PKDμ/PKD antibody (abcam) (ab131460) (1:1000 in WB)

Tissue samples were separated on 10% or15% SDS gels and run at 90 V for 20 minutes following by 125 V for 90 minutes. Thereafter WB was performed to measure the amount and phosphorylation of PKD and HSP27. Following SDS-PAGE, proteins were transferred to polyvinylidene difluoride (PVDF) membranes (Immobilon-P 0.45 µm; Merck Millipore, Burlington, MA, USA). Blots were pre-incubated with 3% bovine serum albumin in Tween Tris-buffered saline (TTBS; containing: 10 mM Tris-HCl; pH 7.6; 75 mM NaCl; 0.1% Tween; all from Sigma-Aldrich) for 1 h at room temperature. Then, blots were incubated overnight at 4°C with the primary antibodies (see above). After washing with TTBS, primary antibody binding was visualized using secondary horseradish peroxidase-labelled, goat anti-rabbit antibody (DakoCytomation, Glostrup, Denmark; dilution 1:10,000) and enhanced chemiluminescence (ECL Western blotting detection; Amersham Biosciences). WB signals were visualized using the LAS-4000 Image Reader and analysed with Multi Gauge V3.2 software (both from FUJIFILM Corp, Minato, Tokyo, Japan). We used GAPDH (Cell Signaling Technology, 1:2000) for comparison of protein load. Finally, signals obtained from the amount and phosphorylation were normalized to signals obtained from GAPDH stains referring to the entire protein amount

transferred. Amount and phosphorylation were expressed in relative numbers, CTRL samples were set to 100%.

CaMKII expression and activity

CaMKII expression was determined using imunoblot (see above; CaMKIIδ from ThermoFisher Scientific, 1:1000) and CaMKII activity ny nonradioactive kinase activity-assay kit (CycLex).

Immunofluorescence imaging

Frozen LV unfixed slides (n=3/group) were air-dried for 10 min and fixed in 10% acetone in phosphate buffered saline (PBS; Sigma-Aldrich). After washing 3 times in PBS for 5 min, tissue was blocked in 5% bovine serum albumin (Sigma-Aldrich) in PBS for 1 h at room temperature. After further washing 3 times in PBS for 5 min, fixed slides were dual-stained with anti-PKD (Sigma-Aldrich; dilution 1:200) or Anti-Phospho-HSP27 (Ser 82) (Cell Signaling Technology; 1:50) and anti-α-actinin (sarcomere; Sigma-Aldrich; dilution 1:400) overnight at 4°C. After washing in PBS, slides were subsequently incubated overnight with secondary antibodies: fluorescein (FITC) anti-mouse (Rockland Immunochemicals Inc, Limerick, PA, USA; dilution 1:300) and Cy3 anti-rabbit (Jackson ImmunoResearch Laboratories Inc, West Grove, PA, USA; dilution 1:100). After multiple washings slides were covered and sealed by Mowiol mounting medium and ultrathin glass coverslips (Thermo Fisher Scientific). Immuno-stained samples were analysed by confocal laser scanning microscopy (Nikon Eclipse Ti-E Inverted Microscope System; Nikon Instruments, Nikon Corp, Shinagawa, Tokyo, Japan). Immunofluorescence imaging was processed equally among groups.

Electron microscopy (EM)

A small piece from a frozen LV was cut and fixed in a 0.1 M PBS buffered fixative containing 4% paraformaldehyde (Sigma Aldrich, St.Louis, MO, USA) and 15% picric acid (Sigma Aldrich, St.Louis, MO, USA) overnight 4°C on shaker. After washing the tissues 3 times in 0.1 M PBS, and blocking with 20% normal goat serum (NGS) in PBS for 1 h, the primary antibody against PKD (Abcam) and HSP27 (Abcam) (1:200) was used in a blocking solution (2% NGS in 0.1 M

PBS) overnight 4°C on shaker. After 3 times washing in 0.1 M PBS the tissues were exposed to 1.4 nm nanogold conjugated anti-rabbit and anti-mouse secondary antibodies (Nanoprobes, NY, USA) (1:100) in the blocking solution overnigh 4°C on shaker. After 3 times washing in 0.1 M PBS the tissues were fixed in 1% glutaraldehyde in PBS for 10 minutes on room temperature. The tissues were washed 3 times in 0.1M PB and 2 times in distillated water, then silver enhancement were performed with HQ silver kit (Nanoprobes, NY, USA). After the enhancement, tissues were washed 2 times with distillated water and 3 times with 0.1M PB. Then the blocks were treated with 0.5% osmium-tetroxid, dehydrated in grading series of ethanol and in propylene oxid and embedded into DURCUPAN™ ACM resin (Sigma Aldrich, St.Louis, MO, USA). Sections of 50 nm were cut, counterstained with UranyLess (Electron Microscopy Sciences, Hatfield, PA, USA) for 10 minutes and investigated using Zeiss LEO 910 electron microscope.

Statistics

Values are given as mean±SEM. Statistically significant differences were tested using Bonferroni adjusted t test unpaired or paired Student t test, with $P<0.05$ considered significant.

References

Agah, R., Frenkel, P.A., French, B.A., Michael, L.H., Overbeek, P.A., and Schneider, M.D. (1997). Gene recombination in postmitotic cells. Targeted expression of Cre recombinase provokes cardiac-restricted, site-specific rearrangement in adult ventricular muscle in vivo. *J Clin Invest* 100**,** 169-179.

Drexler, H.C., Ruhs, A., Konzer, A., Mendler, L., Bruckskotten, M., Looso, M., Gunther, S., Boettger, T., Kruger, M., and Braun, T. (2012). On marathons and Sprints: an integrated quantitative proteomics and transcriptomics analysis of differences between slow and fast muscle fibers. *Mol Cell Proteomics* 11**,** M111 010801.

Fielitz, J., Kim, M.S., Shelton, J.M., Qi, X., Hill, J.A., Richardson, J.A., Bassel-Duby, R., and Olson, E.N. (2008). Requirement of protein kinase D1 for pathological cardiac remodeling. *Proc Natl Acad Sci U S A* 105**,** 3059-3063.

Hamdani, N., Franssen, C., Lourenco, A., Falcao-Pires, I., Fontoura, D., Leite, S., Plettig, L., Lopez, B., Ottenheijm, C.A., Becher, P.M., Gonzalez, A., Tschope, C., Diez, J., Linke, W.A., Leite-Moreira, A.F., and Paulus, W.J. (2013a). Myocardial titin hypophosphorylation importantly contributes to heart failure with preserved ejection fraction in a rat metabolic risk model. *Circ Heart Fail* 6**,** 1239-1249.

Hamdani, N., Krysiak, J., Kreusser, M.M., Neef, S., Dos Remedios, C.G., Maier, L.S., Kruger, M., Backs, J., and Linke, W.A. (2013b). Crucial role for Ca2(+)/calmodulin-dependent protein kinase-II in regulating diastolic stress of normal and failing hearts via titin phosphorylation. *Circ Res* 112**,** 664-674.

Kim, M.S., Fielitz, J., Mcanally, J., Shelton, J.M., Lemon, D.D., Mckinsey, T.A., Richardson, J.A., Bassel-Duby, R., and Olson, E.N. (2008). Protein kinase D1 stimulates MEF2 activity in skeletal muscle and enhances muscle performance. *Mol Cell Biol* 28**,** 3600-3609.

Konzer, A., Ruhs, A., Braun, T., and Kruger, M. (2013). Global protein quantification of mouse heart tissue based on the SILAC mouse. *Methods Mol Biol* 1005**,** 39-52.

Kruger, M., Moser, M., Ussar, S., Thievessen, I., Luber, C.A., Forner, F., Schmidt, S., Zanivan, S., Fassler, R., and Mann, M. (2008). SILAC mouse for quantitative proteomics uncovers kindlin-3 as an essential factor for red blood cell function. *Cell* 134**,** 353-364.

Lundby, A., Secher, A., Lage, K., Nordsborg, N.B., Dmytriyev, A., Lundby, C., and Olsen, J.V. (2012). Quantitative maps of protein phosphorylation sites across 14 different rat organs and tissues. *Nat Commun* 3**,** 876.

Olsen, J.V., Blagoev, B., Gnad, F., Macek, B., Kumar, C., Mortensen, P., and Mann, M. (2006). Global, in vivo, and site-specific phosphorylation dynamics in signaling networks. *Cell* 127**,** 635-648.

Rappsilber, J., Mann, M., and Ishihama, Y. (2007). Protocol for micro-purification, enrichment, pre-fractionation and storage of peptides for proteomics using StageTips. *Nat Protoc* 2**,** 1896-1906.

Shevchenko, A., Tomas, H., Havlis, J., Olsen, J.V., and Mann, M. (2006). In-gel digestion for mass spectrometric characterization of proteins and proteomes. *Nat Protoc* 1**,** 2856-2860.

Wisniewski, J.R., Zougman, A., Nagaraj, N., and Mann, M. (2009). Universal sample preparation method for proteome analysis. *Nat Methods* 6**,** 359-362.

Zanivan, S., Gnad, F., Wickstrom, S.A., Geiger, T., Macek, B., Cox, J., Fassler, R., and Mann, M. (2008). Solid tumor proteome and phosphoproteome analysis by high resolution mass spectrometry. *J Proteome Res* 7**,** 5314-5326.

Zanivan, S., Krueger, M., and Mann, M. (2012). In vivo quantitative proteomics: the SILAC mouse. *Methods Mol Biol* 757**,** 435-450.
